# Reduced Levels of Lagging Strand Polymerases Shape Stem Cell Chromatin

**DOI:** 10.1101/2024.04.26.591383

**Published:** 2024-04-29

**Authors:** Jonathan Snedeker, Brendon E. M. Davis, Rajesh Ranjan, Matthew Wooten, Joshua Blundon, Xin Chen

**Affiliations:** 1 Department of Biology, The Johns Hopkins University, Baltimore, MD 21218, USA; 2 Howard Hughes Medical Institute, Department of Biology, The Johns Hopkins University, 3400 North Charles Street, Baltimore, Baltimore, MD 21218, USA; 3 Current address: Fred Hutchinson Cancer Research Center, Seattle, WA 98109-1024, USA

**Keywords:** germline stem cell, cellular differentiation, asymmetric cell division, DNA replication, chromatin fiber, DNA fiber, lagging strand synthesis, replication-dependent histone incorporation

## Abstract

Stem cells display asymmetric histone inheritance while non-stem progenitor cells exhibit symmetric patterns in the *Drosophila* male germline lineage. Here, we report that components involved in lagging strand synthesis, such as DNA polymerase α and δ (Polα and Polδ), have significantly reduced levels in stem cells compared to progenitor cells. Compromising Polα genetically induces the replication-coupled histone incorporation pattern in progenitor cells to be indistinguishable from that in stem cells, which can be recapitulated using a Polα inhibitor in a concentration-dependent manner. Furthermore, stem cell-derived chromatin fibers display a higher degree of old histone recycling by the leading strand compared to progenitor cell-derived chromatin fibers. However, upon reducing Polα levels in progenitor cells, the chromatin fibers now display asymmetric old histone recycling just like GSC-derived fibers. The old *versus* new histone asymmetry is comparable between stem cells and progenitor cells at both S-phase and M-phase. Together, these results indicate that developmentally programmed expression of key DNA replication components is important to shape stem cell chromatin. Furthermore, manipulating one crucial DNA replication component can induce replication-coupled histone dynamics in non-stem cells in a manner similar to that in stem cells.

## One Sentence Summary

Delayed lagging strand synthesis regulates asymmetric histone incorporation.

## Introduction

Regarding metazoan development, an outstanding question is how cells take on distinct fates and have diverse functions even though they derive from one zygote. Cell fate is determined by selectively expressing a subset of the genome at the proper time, in the right place, and at the precise level. The unique gene expression program for each cell type is typically regulated by the epigenetic mechanisms, which refer to chromatin changes without alteration of the DNA sequences (1–3). Epigenetic mechanisms comprise DNA methylation, histone modifications, histone variants, as well as non-coding RNAs, among others. However, except for DNA methylation, how the epigenetic information is transferred through the active cell cycle in multicellular organisms remains largely unclear (4). Notably, these mechanisms could not only be responsible for maintaining epigenetic memory but also allow for epigenetic changes to diversify cell fates, which are essential for development, homeostasis and regeneration (5–7). One paradigmatic model to study cell fate decision is asymmetric cell division (ACD), through which one mother cell gives rise to two distinct daughter cells. Upon ACD, the genetic codes inherited by the two daughter cells are identical, whereas their epigenetic information can vary, allowing them to appear and function differently [reviewed by (8–12)]. Recently, it has been revealed that ACD can be induced by DNA damage in otherwise symmetrically dividing human cells, suggesting that changes on DNA strands *per se* could guide the cell division mode and potentially regulate the epigenetic inheritance pattern (13).

To investigate the histone inheritance pattern in ACD, a tag-switch strategy to differentially label preexisting (old) *versus* newly synthesized (new) histones has been developed and used to study the *Drosophila* adult stem cell systems. These studies reveal that old histones are selectively retained in the self-renewing stem cell, whereas new histones are enriched in the differentiating daughter cell during ACDs of male germline stem cells (GSCs) (14, 15) and intestinal stem cells (16). Notably, in the male germline lineage, old and new histones are inherited symmetrically during the symmetric divisions of the progenitor spermatogonial cells (SGs). Asymmetric histone inheritance has been proposed to involve a process with at least three steps: First, old and new histones are asymmetrically incorporated on the replicative sister chromatids, attributed by both strand-specific incorporation and biased replication fork movement, including increased unidirectional and asymmetric bidirectional fork progression in early-stage germ cells (15, 17). Then, the epigenetically distinct sister chromatids are differentially recognized and segregated during mitosis (18), leading to distinct “read-outs” in the resulting two daughter cells, such as their asynchronous S-phase initiation (19) and distinct interchromosomal interactions at a key “stemness” gene (20).

Despite this knowledge, two crucial questions still remain: First, what are the precise molecular mechanisms that ensure asymmetric histone incorporation at the individual replication forks? A series of studies have extensively explored the roles of DNA replication components in establishing the epigenomes in unicellular organisms, such as yeast (21–25), and symmetrically dividing cells, such as cultured mouse embryonic stem cells (26–30) and human cell lines (31, 32). These studies focus on how epigenetic information can be equally partitioned between sister chromatids and inherited symmetrically by the daughter cells [reviewed by (4, 33–36)]. Nevertheless, little is known about this process in asymmetrically dividing cells in multicellular organisms. Studies in mouse development demonstrate that asymmetric inheritance of H3R26me2 (37) or maternal chromosome-bound H3.3 and H3K9me2 (38) are essential for early embryogenesis, in contrast to the negative effects of asymmetric histone inheritance in yeast (39–41) and mouse embryonic stem cells (28, 29), emphasizing the importance to study this phenomenon in an organism- and context-dependent manner. Second, how are these mechanisms regulated in a stage-specific manner within the same adult stem cell lineage, such that histone inheritance is asymmetric in stem cells (e.g., GSCs) but symmetric in progenitor cells (e.g., SGs)? Here, we used the *Drosophila* male germline as a model system to address these questions.

## Results

### Differential expression of the lagging strand-enriched replication components in GSCs

**Figure 1:**
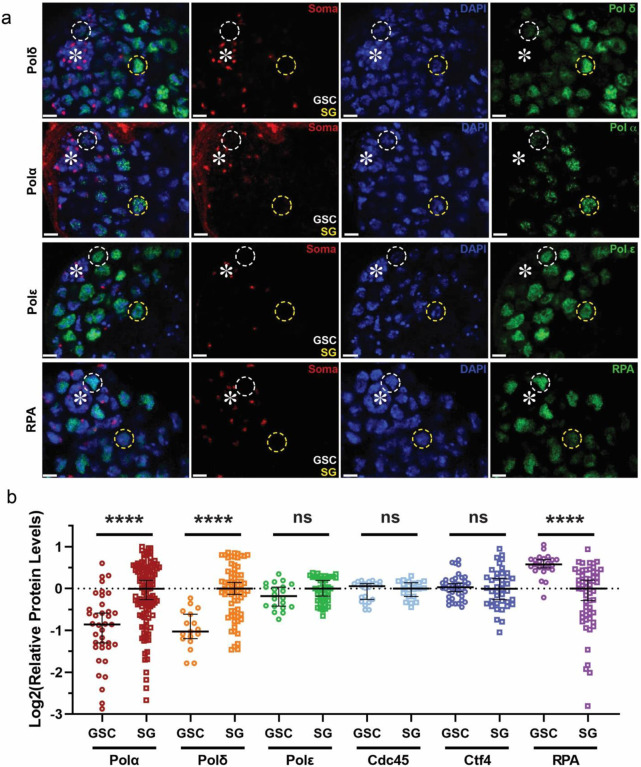
Distinct expression patterns of different replication machinery components in the *Drosophila* male germline stem cell lineage. (**a**) Images of expression of 3×HA tagged endogenous DNA polymerases Polδ, Polα, Polε (see Materials and Methods), as well as the RPA 70-EGFP expressed from a transgene with its own promoter (44). Somatic cell-enriched histone modification H4K20me2/3 (red) (91), DAPI (blue), and the respective replication proteins (green). Both Polδ and Polα show decreased levels in GSCs, while Polε has comparable expression between GSCs and SGs. Contrastingly, RPA is more enriched in GSCs compared to SGs using a transgene under the control of its endogenous regulatory elements (*rpa70>rpa70-EGFP*) (44). Representative GSCs are indicated by the white dotted circle while SGs are indicated by the yellow dotted circle. (**b**) Quantification of the relative expression levels of different replication proteins, using a batch-based normalization to GSCs from the corresponding testis sample followed by log_2_ transformation (Materials and Methods). Medians: GSC Polα log_2_= −0.85 (n= 37), SG Polα log_2_= 0.00 (n= 118); GSC Polδ log_2_= −1.03 (n= 17), SG Polδ log_2_=0.00 (n= 70); GSC Polε log_2_= −0.18 (n= 20), SG Polε log_2_= 0.00 (n= 43); GSC Cdc45 log_2_= 0.06 (n= 21), SG Cdc45 log_2_= 0.00 (n= 21); GSC Ctf4 log_2_= 0.03 (n= 40), SG Ctf4 log_2_= 0.00 (n=40); GSC RPA log_2_= 0.58 (n= 23), SG RPA log_2_= 0.00 (n= 57). Asterisk: hub. Scale bars: 10 μm. All ratios: Median ± 95% Confidence Interval (CI). Mann-Whitney test, ****: *P*< 10^−4^, ns: not significant. See Table S1 for details.

To identify which factors could be responsible for stem cell-specific asymmetric histone inheritance, we performed a candidate gene screen using a series of CRISPR/Cas9-mediated knock-in lines with the hemagglutinin (HA) tag at individual genes that encode distinct key replication machinery components. Intriguingly, the levels of proteins involved in lagging strand synthesis, such as DNA polymerase α and δ (Polα and Polδ), differ significantly between GSCs and SGs, with substantially reduced levels in GSCs compared to SGs (Fig. 1a). In contrast, a key component for leading strand synthesis, DNA polymerase ε (Polε), exhibits comparable levels between GSCs and SGs (Fig. 1a). On the other hand, the single-stranded DNA (ssDNA) binding protein Replication Protein-A 70 (RPA70), the largest subunit of the ssDNA-binding heterotrimeric complex (42, 43), displays the opposite trend with higher RPA levels in GSCs compared to SGs (Fig. 1a), using the RPA70-EGFP fusion protein expressed under the endogenous regulatory elements of the *rpa70* gene (15, 44).

Quantification of these results reveals that GSCs have approximately 51% the levels of Polδ and 58% the levels of Polα as compared to SGs, while Polε levels are comparable between GSCs and SGs (Fig. 1a, b). In contrast, RPA is 1.54-fold more enriched in GSCs than in SGs (Fig. 1a, b). However, other replication machinery components, such as the replication fork progression Cell Division Cycle protein 45 (Cdc45), show no significant difference between GSCs and SGs (Fig. 1b). Another component whose yeast homolog has been shown to have histone chaperoning activities (22), Chromosome Transmission Fidelity 4 (Ctf4), also displays similar levels between GSCs and SGs (Fig. 1b, Fig. S1a).

The significantly reduced levels of lagging strand polymerases in GSCs could lead to relatively delayed lagging strand synthesis, which could result in excessive ssDNA. The higher levels of RPA in GSCs could be responsible for coating and stabilizing ssDNA (45). Moreover, RPA is capable of competing with Polα at ssDNA sites, therefore preventing Polα from binding to and acting on the lagging strand (46–50). Therefore, decreased Polα and increased RPA could cooperatively contribute to measured lagging strand synthesis in the GSCs, which could also underlie the longer cell cycle length of GSCs than SGs, as reported previously (51).

### Reducing Polα levels or inhibiting Polα activities increase old *versus* new histone separation in S-phase nuclei of progenitor cells

**Figure 2:**
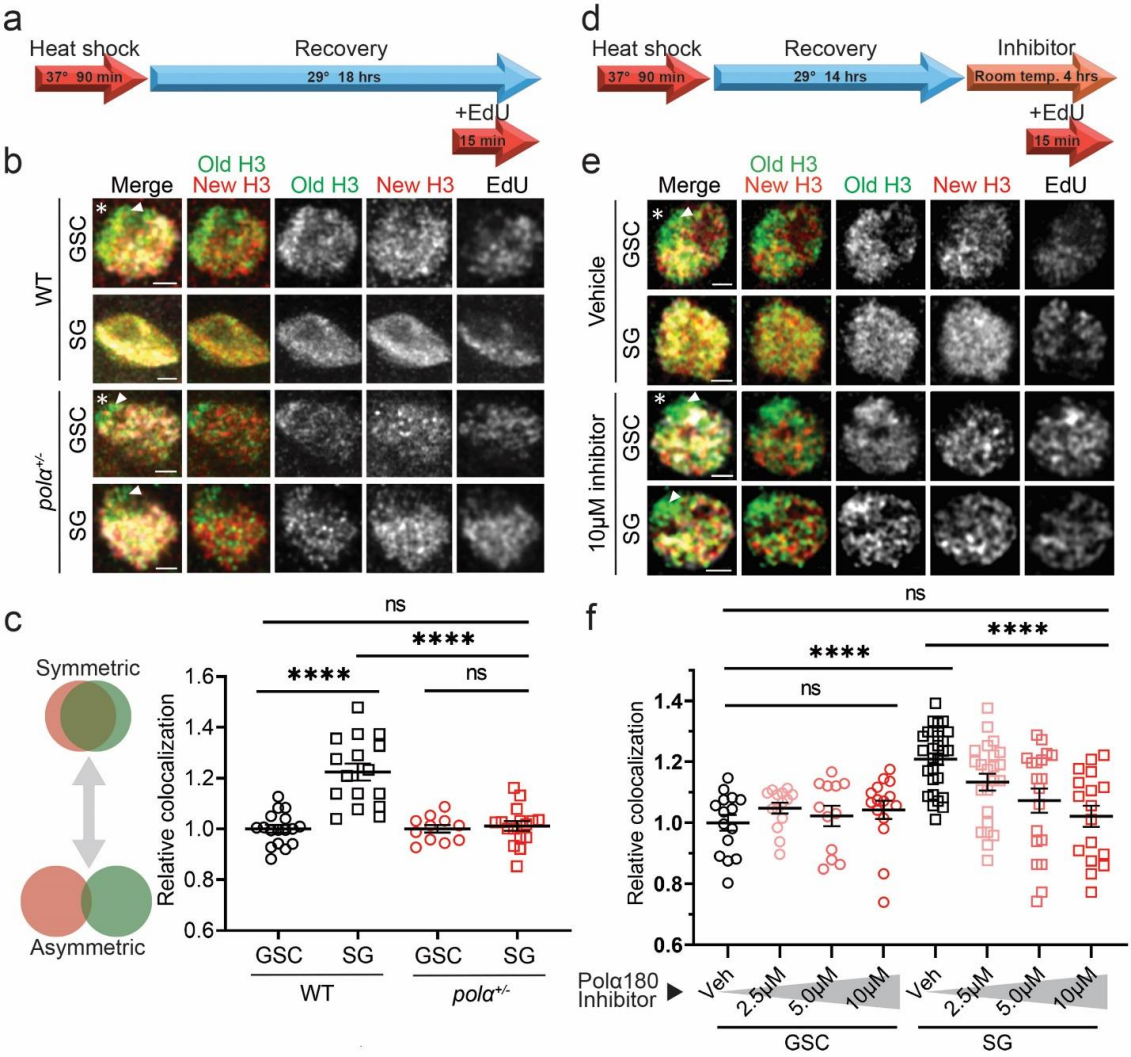
Reducing Polα levels or compromising Polα activities increases old histone *versus* new histone separation in S-phase nuclei of progenitor cells. (**a**) Regime testing old (EGFP) vs. new (mCherry) histone localization pattern following heat shock-induced tag switch. (**b**) Airyscan images of representative control *wild-type* (WT) GSC, WT SG, *polα50*^*+/−*^ GSC, and *polα50*^*+/−*^ SG, respectively, in S-phase nuclei wherein nucleoplasmic histones are largely washed off using a stringent clearance buffer. In all merged images: old H3 (green), new H3 (red), as well as EdU (white), Arm (not shown but used as hub marker). Asterisk: hub. Scale bars: 1 μm. (**c**) Quantification of the correlation between old H3 and new H3 signals in S-phase nuclei using a batch-based normalization to control GSCs (Materials and Methods): WT GSC= 1.00± 0.06 (n= 17), WT SG= 1.90± 0.13 (n= 16), *polα50*^*+/−*^ GSC= 1.00± 0.06 (n= 11), *polα50*^*+/−*^ SG= 1.05± 0.08 (n= 16). See Table S3 for details. All images and quantifications for SGs use the 4-cell SGs. (**d**) Regime testing old vs. new histone localization pattern in response to Polα180 (or Pol A1) inhibitor. (**e**) Airyscan images of representative GSCs and SGs treated with vehicle or Polα180 inhibitor for four hours prior to clearance buffer treatment and fixation. Arrowheads in (**b**) and (**e**): unreplicated regions are enriched with old H3 but depleted with new H3 and EdU labeling. (**f**) Quantification of the correlation between old H3 and new H3 signals in S-phase nuclei following inhibitor treatment using a batch-based normalization to vehicle-treated GSCs (Materials and Methods): Vehicle GSC= 1.00 ± 0.03 (n= 15), 2.5μM GSC= 1.05 ± 0.02 (n= 14), 5.0μM GSC= 1.02 ± 0.03 (n= 12), 10μM GSC= 1.04 ± 0.03 (n= 15); Vehicle SG= 1.21 ± 0.02 (n= 28), 2.5μM SG= 1.13 ± 0.03 (n= 23), 5.0μM SG= 1.07 ± 0.04 (n= 19), 10μM SG= 1.02 ± 0.04 (n= 17). See Table S4 for details. All images and quantifications for SGs use the 4-cell SGs. All ratios: Mean ± Standard Error of the Mean (SEM). Mann-Whitney test, ****: *P*< 10^−4^, ns: not significant.

Based on the above observation, we hypothesize that relatively slow lagging strand synthesis could bias old histone recycling to the finished leading strand at individual replication forks, serving as a key molecular mechanism underlying asymmetric histone incorporation in GSCs. To investigate this hypothesis, we first examined the distribution of old *versus* new histones in intact nuclei using a dual-color system to label old H3 with EGFP and new H3 with mCherry in the male germline (15, 19). To avoid any possible complications caused by non-chromatin-bound histones, we used a stringent clearance buffer that has been shown to effectively remove free histones in the nucleus (19, 52, 53). This strategy, together with high spatial resolution Airyscan microscopy (15, 54), allows us to visualize separable old H3-EGFP *versus* new H3-mCherry enriched regions in the control *wild-type* (WT) GSCs at S-phase, labeled with a pulse of thymidine analog 5-ethynyl-2´-deoxyuridine (EdU) (Fig. 2a, b). In contrast, the degree of separation between old and new H3 is less in WT SGs than that in WT GSCs during S-phase (Fig. 2b). Quantification using a relative Pearson colocalization measurement (16, 55, 56) reveals significantly higher degree of colocalization between old H3-EGFP and new H3-mCherry in WT SGs than in WT GSCs (Fig. 2c), consistent with asymmetric incorporation of old *versus* new histones in S-phase WT GSCs.

We next asked whether compromising lagging strand synthesis in SGs could recapitulate GSC-like features, such as separable old *versus* new histones in the S-phase nuclei. Since replication components are essential for animal survival and cell cycle progression, we sought to compromise lagging strand polymerases without causing cell cycle arrest or cell death, which does occur in strong loss-of-function homozygous or in RNAi knockdown germ cells (data not shown). However, by using a null allele of the *pol*α*50* gene (Materials and Methods), which encodes the DNA Primase Subunit 1 (or Prim1), we generated *pol*α*50*^*+/−*^ heterozygotes. We were able to maintain viable flies with no detectable systematic phenotypes. Intriguingly, when the Primase levels are reduced in *pol*α*50*^*+/−*^ males, S-phase SGs display much more separable patterns between old and new H3, to a level indistinguishable from WT GSCs as well as *pol*α*50*^*+/−*^ GSCs (Fig. 2b, c).

In addition to this genetic approach, we tried a pharmacological strategy with a Polα inhibitor that prevents the DNA binding ability and primer elongation activity of DNA Polymerase α subunit 1 (PolA1 or Polα180, Fig. S2a) (57). At a high concentration (e.g., 100μM), this inhibitor completely blocks DNA replication, indicated by the absence of EdU incorporation in different staged germ cells (data not shown). However, when using this inhibitor at a relatively low concentration (e.g., 10μM), normal DNA replication could proceed with proper EdU incorporation compared to the control sample treated with vehicle (Fig. 2d, Fig. S2b–c). With this inhibitor treatment, SGs exhibit separable old *versus* new H3 patterns similar to those detected in the *pol*α*50*^*+/−*^ cells (inhibitor SG in Fig. 2e vs. *pol*α*50*^*+/−*^ SG in Fig. 2b). The inhibitor-treated SGs show more separation than the vehicle-treated SGs, and display patterns comparable to either inhibitor-treated GSCs or vehicle-treated GSCs (Fig. 2e). Quantifications further reveal that this inhibitor induces old *versus* new H3 separation in SGs in a dosage-dependent manner, but it causes insignificant changes in GSCs regardless of the concentration (Fig. 2f). Notably, the presence of intermediate histone separation patterns at decreasing concentrations of inhibitor (e.g., 5.0μM and 2.5μM) indicate that the asymmetric histone incorporation pattern is tunable and scales to the inhibition of Polα. Additionally, because GSCs are relatively unaffected, we hypothesize that these cells are at the maximum of histone asymmetry and thus cannot be made more asymmetric by compromising Polα.

Moreover, in all imaged nuclei undergoing DNA synthesis, unreplicated regions are enriched with old H3 but devoid of new H3 as well as EdU labeling (arrowheads in Fig. 2b and Fig. 2e), confirming that actively replicating regions are coupled with new H3 incorporation. Together, these data in intact S-phase nuclei demonstrate that reducing primase levels or inhibiting Polα activity are each sufficient to induce separable old *versus* new histone incorporation in S-phase SGs, to a degree indistinguishable from that in GSCs.

### Reducing Polα levels enhances asymmetric old histone incorporation at the replication fork in S-phase progenitor cells

Next, in order to directly visualize the dynamic histone incorporation patterns at the actively replicating regions, a short pulse of EdU was introduced in combination with a single-molecule chromatin fiber technique (15, 17). To precisely label chromatin fibers derived from GSCs *versus* non-stem progenitor SGs, we paired the Gal4 transcription activator controlled by the early germline-specific *nanos* driver (*nos-Gal4ΔVP16*) (58) with the Gal80 transcription repressor under the control of the *bag of marbles* promoter (*bam-Gal80*), which turns on expression from 2-cell to late stage SGs (59). This combination restricts the *H3-EGFP* transgene expression almost exclusively in GSCs with some detectable expression in the gonialblasts (GBs) but almost undetectable signals in the SGs (Fig. S1d), which differs from the early-stage germ cell expression pattern driven solely by *nos-Gal4* (60) (Fig. S1b) and late-stage germ cell expression pattern driven solely by *bam-Gal4* (61–63) (Fig. S1c). These germline stage-specific expression patterns are confirmed by quantification using a *H3-EGFP* reporter (Fig. S1e).

**Figure 3:**
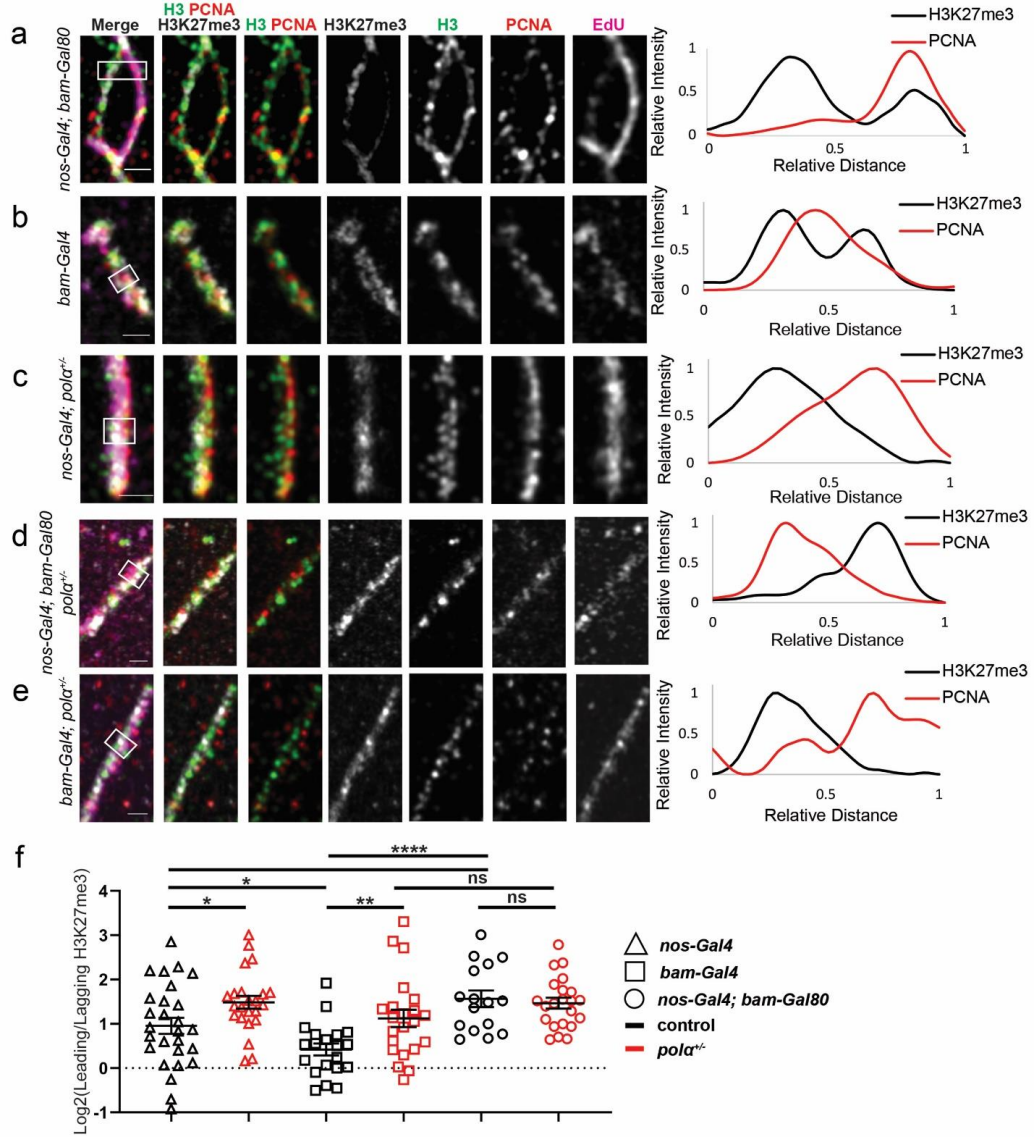
Reducing Polα levels enhances asymmetric old histone recycling at the replication fork in progenitor cells. **(a-e)** Airyscan images of chromatin fibers isolated from testes with the following genotypes: (a) *nos-Gal4****Δ****VP16; bam-Gal80>H3-EGFP*, (b) *bam-Gal4>H3-EGFP*, (**c**) *nanos-Gal4>H3-EGFP; polα50*^*+/−*^, (**d**) *nos-Gal4ΔVP16; bam-Gal80>H3-EGFP; polα50*^*+/−*^, (**e**) *bam-Gal4>H3-EGFP; polα50*^*+/−*^, respectively. In all merged images: H3K27me3 (white), H3-EGFP (green), PCNA (red), and EdU (magenta). All images are accompanied by a line plot showing the distance-dependent H3K27me3 and PCNA signals over the indicated region (white outlined box). (**f**) Quantification of the H3K27me3 signals on chromatin fibers in log_2_ scale: *nanos-Gal4>H3-EGFP*= 0.95± 0.18 (n= 27), *nanos-Gal4>H3-EGFP; polα50*^*+/−*^= 1.49± 0.15 (n= 23), *bam-Gal4>H3-EGFP*= 0.42± 0.14 (n= 20), *bam-Gal4>H3-EGFP; polα50*^*+/−*^= 1.12± 0.20 (n= 22), *nos-Gal4ΔVP16; bam-Gal80>H3-*EGFP= 1.56± 0.19 (n= 16), *nos-Gal4ΔVP16; bam-Gal80>H3-EGFP; polα50*^*+/−*^= 1.66± 0.15 (n= 22). Scale bars: 1 μm. All ratios: Mean± SEM. Mann-Whitney test, ****: *P*< 10^−4^, **: *P*< 0.01, *: *P*< 0.05, ns: not significant. See Table S6 for details.

Using the *H3-EGFP* reporter with different drivers, we labeled chromatin fibers derived from early-stage germ cells including GSCs (*nos>H3-EGFP*), from very early-stage germ cells enriched with almost exclusive GSCs (*nos-Gal4ΔVP16; bam-Gal80*>*H3-EGFP*), and from late-stage SGs (*bam*>*H3-EGFP*). We then explored old histone recycling patterns at the H3-EGFP-labeled and EdU-positive chromatin fibers using the old H3-enriched H3K27me3 histone modification (31, 64, 65). We also distinguished the stranded-ness with the lagging strand-enriched component Proliferating Cell Nuclear Antigen (PCNA) (15, 66). Together, chromatin fibers carrying all four markers (i.e., H3-EGFP, EdU, anti-H3K27me3, and anti-PCNA) were analyzed using super-resolution Airyscan microscopy. While *nos>H3-EGFP*-labeled chromatin fibers show a relatively wide distribution of H3K27me3 between replicative sister chromatids (Fig. S3a, b) with an overall biased distribution toward the PCNA-depleted leading strand (Fig. 3f, Fig. S3c), *nos-Gal4ΔVP16; bam-Gal80*>*H3-EGFP*-labeled chromatin fibers show consistently more asymmetric H3K27me3 distribution toward the leading strand (Fig. 3a, f, Fig. S3c). In contrast, *bam*>*H3-eGFP*-labeled fibers display a more symmetric H3K27me3 distribution pattern (Fig. 3b, f, Fig. S3c). Notably, previous reports using an imaging-based proximity ligation assay in intact nuclei (67, 68) demonstrate that new histones have a substantial lagging strand preference in GSCs but not in SGs (15), consistent with the results shown here.

To quantify old histone incorporation patterns, we used H3K27me3 as a proxy for old histones and plotted its ratio on the PCNA-depleted leading strand to the PCNA-enriched lagging strand (log_2_ ratios in Fig. 3f, Fig. S3c–d). The *nos-Gal4ΔVP16; bam-Gal80*-labeled and the *bam-*labeled chromatin fibers are not only statistically distinguishable from each other (*P*< 10^−4^, Fig. 3f), but also statistically different from the *nos*-labeled group (*P*< 0.05, Fig. 3f). Interestingly, combining the *nos-Gal4ΔVP16; bam-Gal80*-labeled and *bam*-labeled groups *in silico* generates a data set indistinguishable from the *nos*-labeled group (Fig. S3c), suggesting that the heterogeneity of both GSC-derived and SG-derived fibers could underlie the detected H3K27me3 variation among the *nos*-labeled chromatin fibers.

Furthermore, we found that the *nos*-labeled fibers from *pol*α*50*^*+/−*^ testes show more asymmetric H3K27me3 distribution toward the leading strand than the *nos*-labeled fibers from the control (Fig. 3c, f). Consistently, the *nos*-labeled fibers from heterozygotes of the *polα180* gene, which encodes DNA Polymerase α subunit 1 (or PolA1), also exhibit a more asymmetric H3K27me3 distribution pattern toward the leading strand than those from the control (Fig. S3d–e). In contrast, compromising Polα50 has little effect on *nos-Gal4ΔVP16; bam-Gal80*-labeled chromatin fibers (Fig 3d, f). Notably, *bam>H3-eGFP*-labeled chromatin fibers display significantly more asymmetric patterns in the *pol*α*50*^*+/−*^ samples than in the control (Fig 3e, f), in accordance with the results shown in intact S-phase nuclei (Fig. 2b, c). Together, these results demonstrate that compromising Polα affects SGs with normally high levels of Polα (i.e., *bam-*labeled chromatin fibers in Fig. 3 and intact SG nuclei in Fig. 2) more than GSCs that already have low levels of Polα (i.e., *nos-Gal4ΔVP16; bam-Gal80*-labeled chromatin fibers in Fig. 3 and intact GSC nuclei in Fig. 2). It is likely that reducing Polα levels below a certain threshold cannot further increase histone asymmetry, but reducing Polα from relatively high levels (i.e., SG-like) to relatively low levels (i.e., GSC-like) is sufficient to enhance asymmetric old histone recycling at the replication fork.

Finally, to test whether RPA also contributes to asymmetric histone incorporation, we overexpressed the *rpa70* cDNA using *nos-Gal4* (*nos>rpa70-HA)*. Likewise, the overexpression of RPA70 results in enhanced asymmetric H3K27me3 incorporation at the replicative regions, indicating that increased levels of RPA lead to enhanced asymmetric old histone recycling (Fig. S3d, f). Notably, these results are consistent with the previous report that using the *rpa-70>rpa-70-EGFP* line, where the transgenic RPA-70-EGFP fusion protein is under the control of the endogenous *rpa-70* regulatory elements and represents a slight overexpression condition. Under this condition, an average of 3.2-fold leading strand biased H3K27me3 asymmetry is detected, more than the control line which shows an average of 2.0-fold leading strand biased H3K27me3 asymmetry (15). These effects could be attributed to the previously reported competing roles of RPA in preventing Polα from binding to the lagging strand (46–50). In summary, the chromatin fiber results demonstrate that SGs with relatively high levels of Polα and low levels of RPA can be induced to have GSC-like asymmetric old histone incorporation at the replicative regions by reducing Polα levels or by enhancing RPA expression.

### Reducing Polα levels induces differential condensation of old histone- *versus* new histone-enriched regions in M-phase progenitor cells

**Figure 4:**
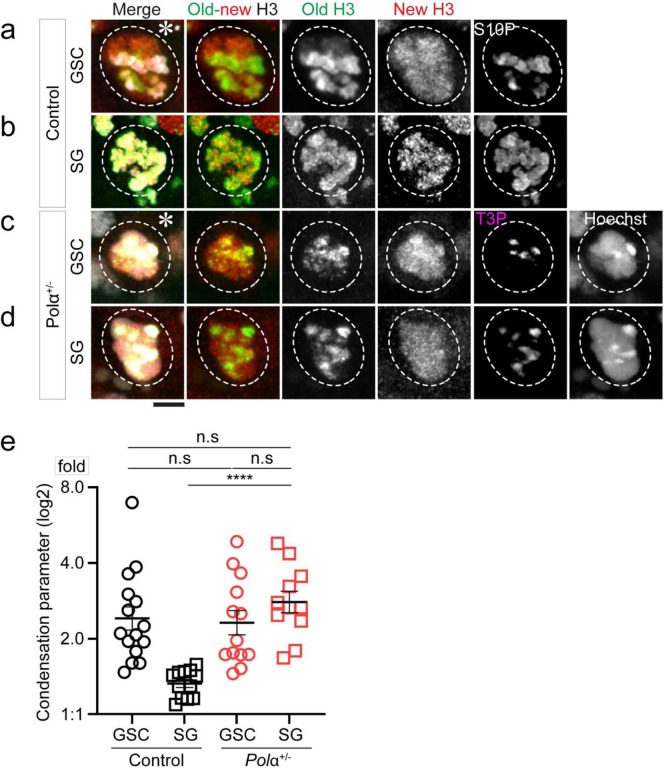
Reducing Polα levels induces differential condensation of old H3 *versus* new H3 enriched regions in M-phase progenitor cells. (**a-b**) Representative images of: (**a**) an M-phase GSC showing more compact old H3-enriched regions than new H3-enriched regions (positive with a mitotic marker anti-H3S10ph, H3S10P or S10P (19); (**b**) an M-phase 8-cell SG showing equally compact old H3-enriched and new H3-enriched regions (positive with S10P) in the control *wild-type* testes. (**c-d**) Representative images of: (**c**) an M-phase GSC and (**d**) an M-phase 8-cell SG in the *pola50*^*+/−*^ testes, both showing more compact old H3-enriched regions than new H3-enriched regions (positive with a mitotic marker anti-H3T3ph, H3T3P or T3P(92). (**e**) Compaction index in log_2_ scale: Control GSC= 1.27± 0.15 (n=15), Control 8-cell SG= 0.41± 0.05 (n=12), *polα50*^*+/−*^ GSC= 1.21± 0.15 (n=13), and *polα50*^*+/−*^ 8-cell SG= 1.48± 0.14 (n=11). The control compaction index data are from (19) with permission. See Table S8 for details. All ratios: Mean± SEM. Mann-Whitney test, ****: *P*< 10^−4^, ns: not significant.

It has been reported that old H3- *versus* new H3-enriched chromosomal regions display differential condensation in the M-phase GSCs but overlapping pattern in the M-phase SGs (19). Consistent with previous reports (19, 53), the control GSCs and SGs display marked condensation differences between old H3- and new H3-enriched regions (Fig. 4a, b), while the *pol*α*50*^*+/−*^ SGs (Fig. 4d) show GSC-like (Fig. 4a, c) differential condensation patterns. Here, using a relative chromatin compaction index to measure the differential condensation between old H3- and new H3-enriched regions as reported previously (19, 53), significant difference could be detected between GSCs and SGs in the control testes but not between GSCs and SGs in the *pol*α*50*^*+/−*^ testes (Fig. 4e). Importantly, in the *pol*α*50*^*+/−*^ testes, both GSCs and SGs display similar patterns compared to the control GSCs but significantly distinct patterns compared to the control SGs (Fig. 4e). Collectively, these results demonstrate that by compromising a single lagging strand-enriched component, differential condensation of old H3- *versus* new H3-enriched regions in M-phase cells, a GSC-specific feature, can be recapitulated in the SGs.

### Detectable asynchrony between leading strand and lagging strand syntheses

**Figure 5:**
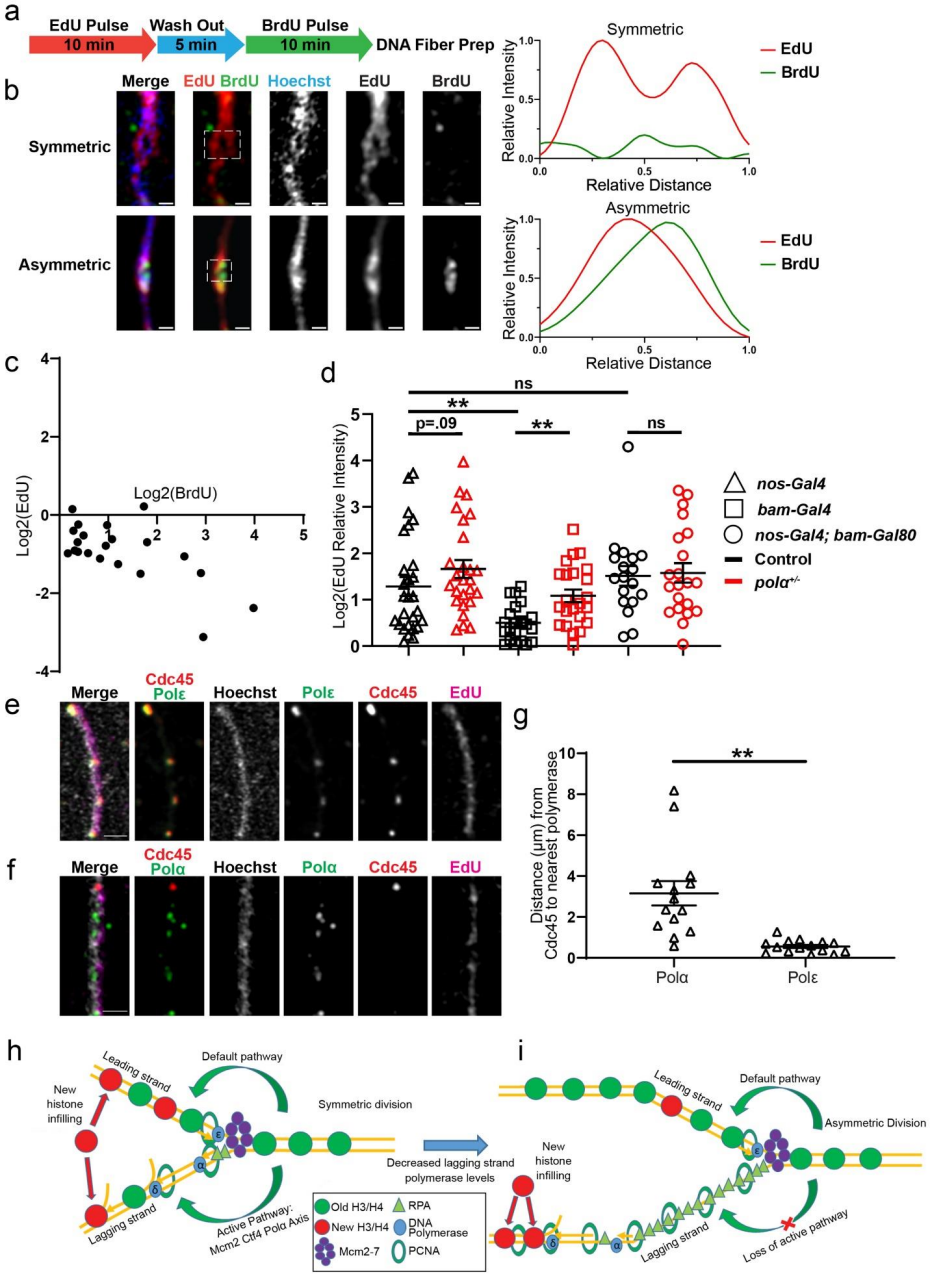
Asynchronous leading strand *versus* lagging strand syntheses. **(a)** Regime with a 10-min EdU pulse followed by a 5-min wash out, then a 10-min BrdU pulse to label DNA fibers (Materials and Methods) (**b**) Airyscan images of DNA fibers: The two line-plots correspond to a representative symmetric replicative region with EdU labeling both strands (white dotted outline on the top panel) and an asymmetric region where EdU and BrdU are on the opposing strands (white dotted outline on the bottom panel). (**c**) Log_2_-scale 2D plot shows the distribution of BrdU and EdU on the DNA fibers with both signals. Most fibers display EdU and BrdU on the opposing DNA strands. See Table S10 for details. (**d**) Quantification of EdU distribution in log_2_-scale, introduced by a 15-minute EdU pulse labeling, on chromatin fibers labeled with H3-EGFP driven by the following drivers without strandedness information: *nanos-Gal4*= 1.29± 0.21 (n= 27), *nanos-Gal4; polα50*^*+/−*^= 1.66± 0.19 (n= 26), *bam-Gal4*= 0.50± 0.09 (n= 21), *bam-Gal4; polα50*^*+/−*^= 1.08± 0.14 (n= 23), *nos-Gal4ΔVP16; bam-Gal80*= 1.52± 0.21 (n= 18), *nos-Gal4ΔVP16; bam-Gal80; polα50*^*+/−*^= 1.58± 0.21 (n= 21). Scale bars: 1 μm. All ratios: Mean± SEM. Mann-Whitney test, **: *P*< 0.01, ns: not significant. See Table S11 for details. (**e-f**) Visualization of delayed lagging strand synthesis: Airyscan image of representative chromatin fibers labeled with endogenous Cdc45-mcherry (red), EdU (magenta), Hoechst (white), along with (**e**) Polε-HA (green) or (**f**) Polα-HA (green). (**g**) Quantification of the distance along chromatin fibers from the center of the Cdc45 signal to the nearest signal of the DNA polymerase: Polα= 3.16± 0.60 μm (n= 14), Polε= 0.56± 0.08 μm (n= 15). Scale bars: 1 μm. All ratios: Mean± SEM. Mann-Whitney test, ****: *P*< 10^−4^. See Table S12 for details. (**h-i**) Models depicting how reducing lagging strand polymerase levels could drive increased histone asymmetry at the replication fork: (**h**) In symmetrically dividing cells, comparable leading strand *versus* lagging strand syntheses give the old histone equal opportunities to be recycled by either strand, based on previous reports(21, 22, 26). (**i**) In asymmetrically dividing cells, reduced levels of lagging strand polymerases lead to measured lagging strand synthesis relative to the leading strand, which results in a temporal difference and biases old histone recycling by the leading strand, whereas new histones infill to the lagging strand.

Next, to measure the leading *versus* lagging strand syntheses in the early-stage germline, we attempted to directly visualize these processes using active incorporation of nucleotide analogs. Previously, it has been shown that the syntheses of the two DNA strands can be discontinuous where the leading and lagging strand polymerases are not tightly coupled in *E. coli* (69) or when applying PolA1 inhibitor in cultured human cells (70). Recently, it has been reported that the temporal differences in replicating leading strand *versus* lagging strand biases old histone incorporation by the strand more closely coupled to the replication fork progression in *S. cerevisiae* (71). To investigate whether this temporal difference exists and is detectable in the *Drosophila* testes, we investigated whether leading strand *versus* lagging strand syntheses can be differentially labeled, using distinct nucleotide analogs introduced in a sequential order [e.g., a short pulse of EdU followed by a short pulse of Bromodeoxyuridine (BrdU), Fig. 5a]. Using this regime, DNA fibers where both strands are co-labeled with just one nucleotide (e.g., EdU) should represent regions where both strands are replicated within the time window of the EdU pulse (an example is shown in the top panel of Fig. 5b). However, DNA fibers with EdU and BrdU on opposing strands likely capture the uncoupled syntheses of the two strands (an example is shown in the bottom panel of Fig. 5b). Indeed, the DNA fibers derived from the apical testis tips display the latter pattern in approximately 40% of the fibers (Fig. S4a). On average, DNA fibers carrying both EdU and BrdU display a 2.35-fold BrdU enrichment toward one strand whereas a 1.91-fold EdU enrichment toward the opposing strand (Fig. 5c, Fig. S4b).

On the other hand, when a single short EdU pulse is introduced on early germline-derived chromatin fibers where strandedness can be determined, 52% of them display a strong bias (>2-fold) toward one of the two strands, with 79% of them displaying strong asymmetry toward the lagging strand (Fig. S4c–d), likely due to a longer time for synthesizing the lagging strand and thus a higher opportunity for the lagging strand to be labeled (Fig. S4e). Consistently, PCNA signals often display asymmetric distribution on early germline-derived chromatin fibers, along with EdU, toward the H3K27me3-depleted lagging strand (Fig. S4d, Fig. 3a, and Fig. S3a).

To further test whether delayed lagging strand synthesis is mechanistically responsible for asymmetric histone incorporation, we analyzed the EdU signals of all chromatin fibers that have been analyzed for the H3K27me3 patterns in Figure 3. Indeed, very early-stage germline-derived (*nanos-Gal4ΔVP16; bam-Gal80*) chromatin fibers show a high degree of asymmetric EdU patterns, while the late-stage germline-derived (*bam-Gal4*) chromatin fibers primarily show symmetric EdU distribution between sister chromatids (Fig. 5d, Fig. S4f). Notably, EdU asymmetry is substantially enhanced in the late-stage germline-derived (*bam-Gal4*) chromatin fibers from the *polα50*^*+/−*^ testes than that from the control (*P*< 0.01 in Fig. 5d, Fig. S4f). The small incidence of leading strand- but a large population of lagging strand-biased EdU incorporation (Fig. S4d, f) are consistent with the temporal asynchrony between leading strand and lagging strand syntheses, with the lagging side synthesizing DNA more slowly on average (Fig. S4e). Overall, these results show that compromising Polα is sufficient to increase asymmetric H3K27me3 incorporation by the leading strand, likely by enhancing the temporal asynchrony between sister chromatid syntheses.

Finally, to directly visualize delayed lagging strand synthesis, we used an endogenously tagged *cdc45* gene, resulting in the Cdc45-mCherry fusion protein as a marker for the Cdc45-MCM-GINS (CMG) complexes (72, 73), in order to label actively progressing replication forks. We then performed immunostaining using antibodies against the HA tag to label Polα-HA for lagging strand polymerase and Polε-HA for leading strand polymerase, using the endogenously tagged genes, respectively. Remarkably, while Polε is always tightly associated with Cdc45 at EdU-labeled replicative chromatin fibers (Fig. 5e, g), Polα could be found in tracts extending away from Cdc45 (Fig. 5f, g), indicating that Polα is spatially decoupled from the actively progressing fork. Together, these results demonstrate that the temporal and spatial separation between leading strand and lagging strand syntheses can be visualized by nucleotide analogs introduced at different time points during DNA replication or with different replisome components.

## Discussion

Here, we report a crucial molecular mechanism underlying asymmetric histone incorporation in stem cells. In symmetrically dividing progenitor cells, comparable leading *versus* lagging strand syntheses give the old histone equal opportunities to be recycled by both strands (Fig. 5h), as shown previously (21, 22, 26, 28, 31). In asymmetrically dividing germline stem cells, reduced lagging strand polymerase levels could slow down lagging strand synthesis relative to the leading strand, which results in a pronounced temporal difference. This difference could bias displaced old histone ahead of the fork to be immediately recycled by the leading strand whereas new histones infill to the lagging strand (Fig. 5i). This model is consistent with a previous report that nucleosomes have the priority to be reincorporated by the double-stranded leading strand *in vitro* (74). The increased expression of RPA in stem cells could also facilitate this process (Fig. 5i). This model is consistent with the previous imaging-based results displaying abundant RPA bound to the lagging strand on early-stage germ cell derived chromatin fibers (15). Intriguingly, either reducing the expression of the key lagging strand polymerase or inhibiting its activity is sufficient to induce stem cell-specific asymmetric histone incorporation patterns even in non-stem progenitor cells.

Notably, even though SGs with approximately 50% Primase (i.e., *pola50*^*+/−*^) have S-phase replication-dependent histone incorporation patterns and M-phase differential chromosomal condensation patterns similar to those in GSCs, SGs do not reside in a polarized microenvironment like the “niche” for GSCs. Additionally, there is no evidence that the microtubule organization centers, the centrosomes, have asymemtric activities in the *wild-type* SGs (62, 75). Therefore, these chromatin asymmetries may not result in substantial differences between the two daughter cells resulting from SG symmetric cell division, unlike the asymmetric division of GSCs. Finally, the *pola50*^*+/−*^ SGs seem to undergo terminal differentiation properly, as there are no obvious germline defects detectable in the *pola50*^*+/−*^ males. It is plausible that the molecular features such as the transcriptome of the *pola50*^*+/−*^ SGs remain unchanged or have inconsequential changes, despite the detectable changes of their chromatin structure. This indicates that the epigenome potentiates cell fate change but may not be determinstic for such a decision. Remarkably, the 50% reduction of Primase in heterozygous SGs is analogous to the protein level change detected in WT GSCs (Fig. 1b). Further reduction of Primase will cause cell cycle arrest, contributing to severe defects detected in homozygotes and under significant knockdown conditions (data not shown).

Furthermore, we focus on the Polα-primase complex in this study because it predominantly acts on the lagging strand except the initial priming event on the leading strand and during rare re-priming events at stalled replication forks (47, 76, 77). On the other hand, Polδ could contribute to the synthesis of both the leading strand and the lagging strand (78, 79). Although it is known that Polα (22, 28) and RPA (80) also play a role in chaperoning histones during replication-coupled nucleosome assembly, such activities have been demonstrated using specific mutations at their histone interacting domains. Here, our studies make use of genetic approaches that either compromise function or change expression of the entire proteins. Additionally, the pharmacological method utilizes enzyme inhibitors whose effects are more related to their roles as replication machinery components, such as primer elongation, rather than chaperones. Notably, DNA replication inhibitors are often used to target over-proliferative cancer cells. Indeed, the drug adarotene and its derivative molecule used in this study have been shown to have anti-cancer properties in mice (57, 81, 82). However, the dose used in our studies is much lower than the dose used for cancer therapy to ensure minimal effect on S-phase progression (Fig. S2b–c). Therefore, both genetic and pharmacological approaches emphasize the importance of the control for the optimal Polα level or inhibitor dosage, which needs to be calibrated empirically in different systems.

Finally, DNA replication is fundamentally an inherently asymmetric process wherein the synthesizing processes of the leading strand *versus* the lagging strand are widely divergent. Previous studies have shown examples of uncoupled leading strand *versus* lagging strand syntheses in bacteria and cultured cells (69–71, 83), particularly in cases where Polα or its priming activity is compromised. It has long been recognized that the leading strand *versus* the lagging strand may have the potential to differentially incorporate nucleosomes (84). Intriguingly, the old histone-enriched H3K9me3 has been shown to be recycled by the leading strand at the retrotransposon elements in order to repress their ectopic transcription in S-phase mouse embryonic stem cells (85). Further, it has been shown that DNA replication speed and timing underlie cell fate regulation in mammalian cells, including mouse and human cells (86–89). Here, our results indicate that the inherent asymmetry of DNA replication itself could be utilized to differentially regulate histone incorporation and this process displays stage specificity within an endogenous adult stem cell lineage. These results point to a very exciting possibility that developmentally programmed expression of key DNA replication components could regulate the establishment of distinct epigenomes in a cell type- and stage-specific manner. Given that replication components as well as histone proteins and their respective modifications are highly conserved, exploring how this mechanism may be utilized in other developmental contexts across different multicellular organisms could be a very intriguing research direction (5, 90). These elegant and efficient mechanisms could be used to balance differential *versus* equal epigenome establishment in asymmetrically *versus* symmetrically dividing cells, which could then impact plasticity *versus* fidelity in cell fate decisions during development, homeostasis and tissue regeneration.

## Supplementary Material

1

## References

[1] Allis C. D., Jenuwein T., The molecular hallmarks of epigenetic control. Nat Rev Genet 17, 487–500 (2016). 27346641 10.1038/nrg.2016.59

[2] Goldberg A. D., Allis C. D., Bernstein E., Epigenetics: a landscape takes shape. Cell 128, 635–638 (2007). 17320500 10.1016/j.cell.2007.02.006

[3] Bonasio R., Tu S., Reinberg D., Molecular signals of epigenetic states. Science 330, 612–616 (2010). 21030644 10.1126/science.1191078 PMC3772643

[4] Escobar T. M., Loyola A., Reinberg D., Parental nucleosome segregation and the inheritance of cellular identity. Nat Rev Genet 22, 379–392 (2021). 33500558 10.1038/s41576-020-00312-w PMC8609916

[5] Urban J. A., Ranjan R., Chen X., Asymmetric Histone Inheritance: Establishment, Recognition, and Execution. Annu Rev Genet, (2022). 10.1146/annurev-genet-072920-125226 PMC10054593 35905975

[6] Grewal S. I. S., The molecular basis of heterochromatin assembly and epigenetic inheritance. Mol Cell 83, 1767–1785 (2023). 37207657 10.1016/j.molcel.2023.04.020 PMC10309086

[7] Vouzas A. E., Gilbert D. M., Replication timing and transcriptional control: beyond cause and effect - part IV. Curr Opin Genet Dev 79, 102031 (2023). 36905782 10.1016/j.gde.2023.102031 PMC10035587

[8] Sunchu B., Cabernard C., Principles and mechanisms of asymmetric cell division. Development 147, (2020). 10.1242/dev.167650 PMC7338270 32601056

[9] Venkei Z. G., Yamashita Y. M., Emerging mechanisms of asymmetric stem cell division. J Cell Biol 217, 3785–3795 (2018). 30232100 10.1083/jcb.201807037 PMC6219723

[10] Zion E. H., Chandrasekhara C., Chen X., Asymmetric inheritance of epigenetic states in asymmetrically dividing stem cells. Curr Opin Cell Biol 67, 27–36 (2020). 32871437 10.1016/j.ceb.2020.08.003 PMC7736099

[11] Blanpain C., Fuchs E., Stem cell plasticity. Plasticity of epithelial stem cells in tissue regeneration. Science 344, 1242281 (2014). 10.1126/science.1242281 PMC4523269 24926024

[12] Knoblich J. A., Asymmetric cell division: recent developments and their implications for tumour biology. Nat Rev Mol Cell Biol 11, 849–860 (2010). 21102610 10.1038/nrm3010 PMC3941022

[13] Ferrand J. , Mitotic chromatin marking governs asymmetric segregation of DNA damage. bioRxiv, (2023). 10.1038/s41467-025-56090-8 PMC11739639 39820273

[14] Tran V., Lim C., Xie J., Chen X., Asymmetric division of Drosophila male germline stem cell shows asymmetric histone distribution. Science 338, 679–682 (2012). 23118191 10.1126/science.1226028 PMC3532436

[15] Wooten M. , Asymmetric histone inheritance via strand-specific incorporation and biased replication fork movement. Nat Struct Mol Biol 26, 732–743 (2019). 31358945 10.1038/s41594-019-0269-z PMC6684448

[16] Zion E. H. , Old and newly synthesized histones are asymmetrically distributed in Drosophila intestinal stem cell divisions. EMBO Rep 24, e56404 (2023). 37255015 10.15252/embr.202256404 PMC10328082

[17] Wooten M. , Superresolution imaging of chromatin fibers to visualize epigenetic information on replicative DNA. Nat Protoc 15, 1188–1208 (2020). 32051613 10.1038/s41596-019-0283-y PMC7255620

[18] Ranjan R., Snedeker J., Chen X., Asymmetric Centromeres Differentially Coordinate with Mitotic Machinery to Ensure Biased Sister Chromatid Segregation in Germline Stem Cells. Cell Stem Cell 25, 666–681 e665 (2019). 31564548 10.1016/j.stem.2019.08.014 PMC6842444

[19] Ranjan R. , Differential condensation of sister chromatids acts with Cdc6 to ensure asynchronous S-phase entry in Drosophila male germline stem cell lineage. Dev Cell 57, 1102–1118 e1107 (2022). 35483360 10.1016/j.devcel.2022.04.007 PMC9134767

[20] Antel M. , Interchromosomal interaction of homologous Stat92E alleles regulates transcriptional switch during stem-cell differentiation. Nat Commun 13, 3981 (2022). 35810185 10.1038/s41467-022-31737-y PMC9271046

[21] Yu C. , A mechanism for preventing asymmetric histone segregation onto replicating DNA strands. Science 361, 1386–1389 (2018). 30115745 10.1126/science.aat8849 PMC6597248

[22] Gan H. , The Mcm2-Ctf4-Polalpha Axis Facilitates Parental Histone H3-H4 Transfer to Lagging Strands. Mol Cell 72, 140–151 e143 (2018). 30244834 10.1016/j.molcel.2018.09.001 PMC6193272

[23] Shan Z. , The patterns and participants of parental histone recycling during DNA replication in Saccharomyces cerevisiae. Sci China Life Sci, (2023). 10.1007/s11427-022-2267-6 36914923

[24] Schlissel G., Rine J., The nucleosome core particle remembers its position through DNA replication and RNA transcription. Proc Natl Acad Sci U S A 116, 20605–20611 (2019). 31511420 10.1073/pnas.1911943116 PMC6789558

[25] Li N. , Parental histone transfer caught at the replication fork. Nature 627, 890–897 (2024). 38448592 10.1038/s41586-024-07152-2

[26] Petryk N. , MCM2 promotes symmetric inheritance of modified histones during DNA replication. Science 361, 1389–1392 (2018). 30115746 10.1126/science.aau0294

[27] Escobar T. M. , Active and Repressed Chromatin Domains Exhibit Distinct Nucleosome Segregation during DNA Replication. Cell 179, 953–963 e911 (2019). 31675501 10.1016/j.cell.2019.10.009 PMC6917041

[28] Li Z. , DNA polymerase alpha interacts with H3-H4 and facilitates the transfer of parental histones to lagging strands. Sci Adv 6, eabb5820 (2020). 32923642 10.1126/sciadv.abb5820 PMC7449674

[29] Wenger A. , Symmetric inheritance of parental histones governs epigenome maintenance and embryonic stem cell identity. Nat Genet 55, 1567–1578 (2023). 37666988 10.1038/s41588-023-01476-x PMC10484787

[30] Flury V. , Recycling of modified H2A-H2B provides short-term memory of chromatin states. Cell 186, 1050–1065 e1019 (2023). 36750094 10.1016/j.cell.2023.01.007 PMC9994263

[31] Reveron-Gomez N. , Accurate Recycling of Parental Histones Reproduces the Histone Modification Landscape during DNA Replication. Mol Cell 72, 239–249 e235 (2018). 30146316 10.1016/j.molcel.2018.08.010 PMC6202308

[32] Xu M. , Partitioning of histone H3-H4 tetramers during DNA replication-dependent chromatin assembly. Science 328, 94–98 (2010). 20360108 10.1126/science.1178994

[33] Du W. , Mechanisms of chromatin-based epigenetic inheritance. Sci China Life Sci 65, 2162–2190 (2022). 35792957 10.1007/s11427-022-2120-1 PMC10311375

[34] Serra-Cardona A., Zhang Z., Replication-Coupled Nucleosome Assembly in the Passage of Epigenetic Information and Cell Identity. Trends Biochem Sci 43, 136–148 (2018). 29292063 10.1016/j.tibs.2017.12.003 PMC5805396

[35] Stewart-Morgan K. R., Petryk N., Groth A., Chromatin replication and epigenetic cell memory. Nat Cell Biol 22, 361–371 (2020). 32231312 10.1038/s41556-020-0487-y

[36] Zhang W., Feng J., Li Q., The replisome guides nucleosome assembly during DNA replication. Cell Biosci 10, 37 (2020). 32190287 10.1186/s13578-020-00398-z PMC7066812

[37] White M. D. , Long-Lived Binding of Sox2 to DNA Predicts Cell Fate in the Four-Cell Mouse Embryo. Cell 165, 75–87 (2016). 27015308 10.1016/j.cell.2016.02.032

[38] Wen, Z. D. a. R., Activation of the Maternal Genome Through Asymmetric Distribution of Oocyte-Genome-Associated Histone H3.3. bioRxiv, (2023).

[39] Foltman M. , Eukaryotic replisome components cooperate to process histones during chromosome replication. Cell Rep 3, 892–904 (2013). 23499444 10.1016/j.celrep.2013.02.028

[40] Iida T., Araki H., Noncompetitive counteractions of DNA polymerase epsilon and ISW2/yCHRAC for epigenetic inheritance of telomere position effect in Saccharomyces cerevisiae. Mol Cell Biol 24, 217–227 (2004). 14673157 10.1128/MCB.24.1.217-227.2004 PMC303358

[41] Saxton D. S., Rine J., Epigenetic memory independent of symmetric histone inheritance. Elife 8, (2019). 10.7554/eLife.51421 PMC6850775 31613222

[42] Mitsis P. G., Kowalczykowski S. C., Lehman I. R., A single-stranded DNA binding protein from Drosophila melanogaster: characterization of the heterotrimeric protein and its interaction with single-stranded DNA. Biochemistry 32, 5257–5266 (1993). 8494903 10.1021/bi00070a038

[43] Marton R. F., Thommes P., Cotterill S., Purification and characterisation of dRP-A: a single-stranded DNA binding protein from Drosophila melanogaster. FEBS Lett 342, 139–144 (1994). 8143866 10.1016/0014-5793(94)80488-5

[44] Blythe S. A., Wieschaus E. F., Zygotic genome activation triggers the DNA replication checkpoint at the midblastula transition. Cell 160, 1169–1181 (2015). 25748651 10.1016/j.cell.2015.01.050 PMC4359640

[45] Chen J., Le S., Basu A., Chazin W. J., Yan J., Mechanochemical regulations of RPA’s binding to ssDNA. Scientific Reports 5, 9296 (2015). 25787788 10.1038/srep09296 PMC4365408

[46] Jones M. L., Aria V., Baris Y., Yeeles J. T. P., How Pol alpha-primase is targeted to replisomes to prime eukaryotic DNA replication. Mol Cell 83, 2911–2924 e2916 (2023). 37506699 10.1016/j.molcel.2023.06.035 PMC10501992

[47] Taylor M. R. G., Yeeles J. T. P., The Initial Response of a Eukaryotic Replisome to DNA Damage. Mol Cell 70, 1067–1080 e1012 (2018). 29944888 10.1016/j.molcel.2018.04.022 PMC6024075

[48] Collins K. L., Kelly T. J., Effects of T antigen and replication protein A on the initiation of DNA synthesis by DNA polymerase alpha-primase. Mol Cell Biol 11, 2108–2115 (1991). 1848671 10.1128/mcb.11.4.2108-2115.1991 PMC359898

[49] Huang H. , Structure of a DNA polymerase alpha-primase domain that docks on the SV40 helicase and activates the viral primosome. J Biol Chem 285, 17112–17122 (2010). 20234039 10.1074/jbc.M110.116830 PMC2878050

[50] Weisshart K. , Protein-protein interactions of the primase subunits p58 and p48 with simian virus 40 T antigen are required for efficient primer synthesis in a cell-free system. J Biol Chem 275, 17328–17337 (2000). 10747950 10.1074/jbc.M000717200

[51] Gadre P., Nitsure N., Mazumdar D., Gupta S., Ray K., The rates of stem cell division determine the cell cycle lengths of its lineage. iScience 24, 103232 (2021). 34746698 10.1016/j.isci.2021.103232 PMC8555441

[52] Carroll T. D., Newton I. P., Chen Y., Blow J. J., Nathke I., Lgr5(+) intestinal stem cells reside in an unlicensed G1 phase. J Cell Biol 217, 1667–1685 (2018). 29599208 10.1083/jcb.201708023 PMC5940300

[53] Ranjan R., Chen X., Quantitative imaging of chromatin inheritance using a dual-color histone in Drosophila germinal stem cells. STAR Protoc 3, 101811 (2022). 36386868 10.1016/j.xpro.2022.101811 PMC9640340

[54] Sivaguru M. , Comparative performance of airyscan and structured illumination superresolution microscopy in the study of the surface texture and 3D shape of pollen. Microsc Res Tech 81, 101–114 (2018). 27476493 10.1002/jemt.22732

[55] Kahney E. W. , Characterization of histone inheritance patterns in the Drosophila female germline. EMBO Rep, e51530 (2021). 34031963 10.15252/embr.202051530 PMC8406404

[56] Chandrasekhara C. , A single N-terminal amino acid determines the distinct roles of histones H3 and H3.3 in the Drosophila male germline stem cell lineage. PLoS Biol 21, e3002098 (2023). 37126497 10.1371/journal.pbio.3002098 PMC10174566

[57] Cincinelli R. , Novel adamantyl retinoid-related molecules with POLA1 inhibitory activity. Bioorg Chem 104, 104253 (2020). 32920362 10.1016/j.bioorg.2020.104253

[58] Inaba M., Buszczak M., Yamashita Y. M., Nanotubes mediate niche-stem-cell signalling in the Drosophila testis. Nature 523, 329–332 (2015). 26131929 10.1038/nature14602 PMC4586072

[59] Matias N. R., Mathieu J., Huynh J. R., Abscission is regulated by the ESCRT-III protein shrub in Drosophila germline stem cells. PLoS Genet 11, e1004653 (2015). 25647097 10.1371/journal.pgen.1004653 PMC4372032

[60] Van Doren M., Williamson A. L., Lehmann R., Regulation of zygotic gene expression in Drosophila primordial germ cells. Curr Biol 8, 243–246 (1998). 9501989 10.1016/s0960-9822(98)70091-0

[61] Eun S. H., Shi Z., Cui K., Zhao K., Chen X., A non-cell autonomous role of E(z) to prevent germ cells from turning on a somatic cell marker. Science 343, 1513–1516 (2014). 24675960 10.1126/science.1246514 PMC4040133

[62] Cheng J. , Centrosome misorientation reduces stem cell division during ageing. Nature 456, 599–604 (2008). 18923395 10.1038/nature07386 PMC2712891

[63] Chen D., McKearin D. M., A discrete transcriptional silencer in the bam gene determines asymmetric division of the Drosophila germline stem cell. Development 130, 1159–1170 (2003). 12571107 10.1242/dev.00325

[64] Alabert C. , Two distinct modes for propagation of histone PTMs across the cell cycle. Genes Dev 29, 585–590 (2015). 25792596 10.1101/gad.256354.114 PMC4378191

[65] Lin S., Yuan Z. F., Han Y., Marchione D. M., Garcia B. A., Preferential Phosphorylation on Old Histones during Early Mitosis in Human Cells. J Biol Chem 291, 15342–15357 (2016). 27226594 10.1074/jbc.M116.726067 PMC4946945

[66] Yu C. , Strand-specific analysis shows protein binding at replication forks and PCNA unloading from lagging strands when forks stall. Mol Cell 56, 551–563 (2014). 25449133 10.1016/j.molcel.2014.09.017 PMC4362665

[67] Petruk S. , TrxG and PcG proteins but not methylated histones remain associated with DNA through replication. Cell 150, 922–933 (2012). 22921915 10.1016/j.cell.2012.06.046 PMC3432699

[68] Fenstermaker T. K., Petruk S., Kovermann S. K., Brock H. W., Mazo A., RNA polymerase II associates with active genes during DNA replication. Nature 620, 426–433 (2023). 37468626 10.1038/s41586-023-06341-9 PMC13343614

[69] Graham J. E., Marians K. J., Kowalczykowski S. C., Independent and Stochastic Action of DNA Polymerases in the Replisome. Cell 169, 1201–1213 e1217 (2017). 28622507 10.1016/j.cell.2017.05.041 PMC5548433

[70] Ercilla A. , Physiological Tolerance to ssDNA Enables Strand Uncoupling during DNA Replication. Cell Rep 30, 2416–2429 e2417 (2020). 32075739 10.1016/j.celrep.2020.01.067

[71] Ziane R., Camasses A., Radman-Livaja M., The asymmetric distribution of RNA polymerase II and nucleosomes on replicated daughter genomes is caused by differences in replication timing between the lagging and the leading strand. Genome Res 32, 337–356 (2022). 35042724 10.1101/gr.275387.121 PMC8805712

[72] Bochman M. L., Schwacha A., The Mcm complex: unwinding the mechanism of a replicative helicase. Microbiol Mol Biol Rev 73, 652–683 (2009). 19946136 10.1128/MMBR.00019-09 PMC2786579

[73] Tye B. K., MCM proteins in DNA replication. Annu Rev Biochem 68, 649–686 (1999). 10872463 10.1146/annurev.biochem.68.1.649

[74] Brennan L. D., Forties R. A., Patel S. S., Wang M. D., DNA looping mediates nucleosome transfer. Nat Commun 7, 13337 (2016). 27808093 10.1038/ncomms13337 PMC5097161

[75] Yamashita Y. M., Mahowald A. P., Perlin J. R., Fuller M. T., Asymmetric inheritance of mother versus daughter centrosome in stem cell division. Science 315, 518–521 (2007). 17255513 10.1126/science.1134910 PMC2563045

[76] Arezi B., Kuchta R. D., Eukaryotic DNA primase. Trends Biochem Sci 25, 572–576 (2000). 11084371 10.1016/s0968-0004(00)01680-7

[77] Johansson E., Macneill S. A., The eukaryotic replicative DNA polymerases take shape. Trends Biochem Sci 35, 339–347 (2010). 20163964 10.1016/j.tibs.2010.01.004

[78] Zhou Z. X., Lujan S. A., Burkholder A. B., Garbacz M. A., Kunkel T. A., Roles for DNA polymerase delta in initiating and terminating leading strand DNA replication. Nat Commun 10, 3992 (2019). 31488849 10.1038/s41467-019-11995-z PMC6728351

[79] Johnson R. E., Klassen R., Prakash L., Prakash S., A Major Role of DNA Polymerase delta in Replication of Both the Leading and Lagging DNA Strands. Mol Cell 59, 163–175 (2015). 26145172 10.1016/j.molcel.2015.05.038 PMC4517859

[80] Liu S. , RPA binds histone H3-H4 and functions in DNA replication-coupled nucleosome assembly. Science 355, 415–420 (2017). 28126821 10.1126/science.aah4712

[81] Cincinelli R. , A novel atypical retinoid endowed with proapoptotic and antitumor activity. J Med Chem 46, 909–912 (2003). 12620066 10.1021/jm025593y

[82] Nasr R. R. , ST1926, an orally active synthetic retinoid, induces apoptosis in chronic myeloid leukemia cells and prolongs survival in a murine model. Int J Cancer 137, 698–709 (2015). 25557649 10.1002/ijc.29407

[83] Zwinderman M. R. H. , Deposition Bias of Chromatin Proteins Inverts under DNA Replication Stress Conditions. ACS Chem Biol 16, 2193–2201 (2021). 34592816 10.1021/acschembio.1c00321 PMC8609521

[84] Seidman M. M., Levine A. J., Weintraub H., The asymmetric segregation of parental nucleosomes during chrosome replication. Cell 18, 439–449 (1979). 227608 10.1016/0092-8674(79)90063-1

[85] Li Z. , Asymmetric distribution of parental H3K9me3 in S phase silences L1 elements. Nature, (2023). 10.1038/s41586-023-06711-3 PMC11034792 37938774

[86] Nakatani T. , DNA replication fork speed underlies cell fate changes and promotes reprogramming. Nat Genet 54, 318–327 (2022). 35256805 10.1038/s41588-022-01023-0 PMC8920892

[87] Nakatani T. , Emergence of replication timing during early mammalian development. Nature 625, 401–409 (2024). 38123678 10.1038/s41586-023-06872-1 PMC10781638

[88] Klein K. N. , Replication timing maintains the global epigenetic state in human cells. Science 372, 371–378 (2021). 33888635 10.1126/science.aba5545 PMC8173839

[89] Sima J. , Identifying cis Elements for Spatiotemporal Control of Mammalian DNA Replication. Cell 176, 816–830 e818 (2019). 30595451 10.1016/j.cell.2018.11.036 PMC6546437

[90] Muhlen D., Li X., Dovgusha O., Jackle H., Gunesdogan U., Recycling of parental histones preserves the epigenetic landscape during embryonic development. Sci Adv 9, eadd6440 (2023). 36724233 10.1126/sciadv.add6440 PMC9891698

[91] Hennig W., Weyrich A., Histone modifications in the male germ line of Drosophila. BMC Dev Biol 13, 7 (2013). 23433182 10.1186/1471-213X-13-7 PMC3602674

[92] Xie J. , Histone H3 Threonine Phosphorylation Regulates Asymmetric Histone Inheritance in the Drosophila Male Germline. Cell 163, 920–933 (2015). 26522592 10.1016/j.cell.2015.10.002 PMC4636931

