# Supplementary material for: Reduced Levels of Lagging Strand Polymerases Shape Stem Cell Chromatin
Source: bioRxiv. 2024 Apr 29:2024.04.26.591383. Preprint. [Version 1] doi: 10.1101/2024.04.26.591383 (PMC11092439; doi:10.1101/2024.04.26.591383)
Supplement: 1 [file NIHPP2024.04.26.591383V1-supplement-1.pdf]

bioRxiv preprint doi: https://doi.org/10.1101/2024.04.26.591383; this version posted April 29, 2024. The copyright holder for this preprint (which was not certified by peer review) is the author/funder, who has granted bioRxiv a license to display the preprint in perpetuity. It is made available under aCC-BY 4.0 International license.

Materials and Methods

Fly strains and husbandry. Fly strains were raised on standard Bloomington media. All flies were raised at 25°C unless noted otherwise. The following fly strains were used: *hs-flp* on the X chromosome (Bloomington Stock Center BL-26902), *nos-Gal4 (with VP16)* on the 2nd chromosome (*1*), *nos-Gal4* (*without VP16* or *ΔVP16*) on the 2nd chromosome [(from Yukiko Yamashita, Whitehead Institute, USA) and used in (*2*)], *bam-Gal4* on the 3rd chromosome (*3*), *bam-Gal80* on the 3rd chromosome (from Juliette Mathieu and Jean-René Huynh, Collège de France, France), *UASp-FRT-H3-EGFP-FRT-H3-mCherry* on the 2nd chromosome as reported previously (*4*), *polα50* P-element insertion (BL-27205), *polα180* P-element insertion (BL-31805), *pcna>EGFP-pcna* and *rpa>rpa-EGFP* [from Eric Wieschaus, Princeton University, USA and used in (*5*)].

The *polα50* P-element insertion (BL-27205) was verified by sequencing to be a null allele using the following primers: 5'-AGCTCCAATCGTGTATCTCTCT- 3' (specific to the 5' UTR of the *polα50* gene locus) and 5'-CAATCATATCGCTGTCTCACTC- 3' (specific to the P-element sequences of the EP insertion) were used to amplify the genomic sequences corresponding to the 5' end of the *polα50* gene locus, where the P-element insertion was located based on the Flybase (https://flybase.org/). Sequencing with this pair of primers confirmed that the P-element is inserted at a position nine base pairs downstream of the start codon, resulting in

bioRxiv preprint doi: https://doi.org/10.1101/2024.04.26.591383; this version posted April 29, 2024. The copyright holder for this preprint (which was not certified by peer review) is the author/funder, who has granted bioRxiv a license to display the preprint in perpetuity. It is made available under aCC-BY 4.0 International license.

the new coding sequence 5'-ATGCCCGAAcatgatgaaataacataa (lowercase sequences indicate the P-element insertion). This leads to eight codons followed by a stop codon (underlined). Hence, this allele results in an early stop codon that very likely represents a null *loss-of-function* allele of the *polα50* gene. This allele is not homozygous viable and is maintained as a heterozygous stock over a balancer chromosome. All experiments using the *polα50*$^{+/-}$ background were outcrossing the *polα50*/*Balancer* stock to a *wild-type* stock to have the *polα50* P-element insertion allele over a wild-type chromosome.

Generating knock-in fly strains. Endogenously tagged fly strains were generated by CRISPR-Cas9 with the genome editing service provided by Fungene Inc. (Beijing, China). The knock-in strains encoding the following proteins were generated and used in this study: Cdc45-mCherry (internally tagged between D163 and Q164), Cdc45-3×HA (internally tagged between D163 and Q164), DNA polymerase ε 255kD subunit-3×HA (tagged at the C-terminus), DNA polymerase α 180kD-3×HA (tagged at the C-terminus), DNA polymerase δ-3×HA (tagged at the C-terminus), Ctf4-EGFP (tagged at the C-terminus).

Heat shock scheme. Flies with *UASp-FRT-H3-EGFP-FRT-H3-mCherry* along with any relevant genotypes were crossed with *hs-flp; nanos-Gal4* and raised at 25°C. Within two days of eclosure, adult male flies were transferred to a vial and the vial was submerged underwater at 37°C for 90 minutes. Flies were then recovered at 29°C for 18 hours prior to dissection for experiments, with the exception of experiments using the PolA1 inhibitor, as described below.

bioRxiv preprint doi: https://doi.org/10.1101/2024.04.26.591383; this version posted April 29, 2024. The copyright holder for this preprint (which was not certified by peer review) is the author/funder, who has granted bioRxiv a license to display the preprint in perpetuity. It is made available under aCC-BY 4.0 International license.

Whole mount immunostaining experiments. Immunostaining experiments were performed using standard procedure (*6*). Primary antibodies used were Armadillo (Arm, 1:100; DSHB N2 7A1), Traffic Jam (Tj, 1:100, from Mark Van Doren, Johns Hopkins University, USA), anti-PCNA (1:100; Santa Cruz sc-56), anti-GFP (1:1,000; Abcam ab 13970), anti-HA (1:200; Sigma-Aldrich H3663), anti-mCherry (1:1,000; Invitrogen M11217), anti-H3K27me3 (1:400; Millipore 07-449), anti- H4K20me2/3 (1:400; Abcam ab78517), anti-H3S10ph (1:2000; Cell Signaling Technology 9701), rabbit anti- H3T3ph (1:200, Millipore 05-746R), and anti-BrdU (1:200; Abcam ab6326). BrdU analog was Invitrogen B23151 5-bromo-2′-deoxyuridine (BrdU). Secondary antibodies were the Alexa Fluor-conjugated series (1:1,000; Molecular Probes). Confocal images were taken on the Zeiss LSM800 (with Airyscan mode) with a 63x oil objective lenses or on the Leica SPE with 63x oil immersion lenses.

Quantification of protein levels in the early germline. Images were analyzed using the ImageJ software FIJI. Germline cyst stages were identified using Arm signal to label the two cyst cells encapsulating each cyst. Average intensity values were recorded for the center Z-slice of each cell/nucleus of interest. For germ cells within one cyst, only one germline nucleus from the entire cyst was measured as one data point. For the comparison of protein levels of endogenously tagged proteins, immunostaining signals in GSCs, 4-cell and 8-cell SGs were measured, and a background was subtracted using the post-mitotic hub cells, which are devoid of signals from any of these replication components. Signal intensity from 4-cell and 8-cell SGs were then normalized to the average intensity of GSCs from the same batch of testes. For the batch-based normalization, within one experimental batch, each data point is normalized to the average of WT GSCs in this corresponding batch. To compare data among different batches, the resulting

bioRxiv preprint doi: https://doi.org/10.1101/2024.04.26.591383; this version posted April 29, 2024. The copyright holder for this preprint (which was not certified by peer review) is the author/funder, who has granted bioRxiv a license to display the preprint in perpetuity. It is made available under aCC-BY 4.0 International license.

values were then used to calculate the relative amount of GSC protein level to SG protein level (set to 1 to facilitate comparison), and plot on a $\log_2$ scale (Fig. 1b). The dataset shown in Figure 1b are from germ cells at each corresponding differentiation stages (Table S1). We also labeled S-phase germ cells using a EdU pulse and quantified them separately. The results using S-phase germ cells were similar to those using germ cells without distinguishing S-phase from G2-phase (data not shown).

For the comparison of the stage-specificity of each driver or driver combination, *nanos-Gal4* by itself, *nos-Gal4ΔVP16; bam-Gal80* combination, or *bam-Gal4* by itself was crossed to the *UASp-FRT-H3-EGFP-FRT-H3-mCherry* transgene without *hs-flp*. The EGFP signals reflecting the relative strength of each driver or driver combination were quantified in the corresponding germline cyst stages, identified using Arm to label the two encapsulating cyst cells. The central slice of a representative nucleus was taken for each cyst measured as one data point. The cytoplasmic space was used as a background for subtraction. The EGFP signals were normalized to the stage with the highest relative signal intensity: For *nanos-Gal4* by itself, all quantifications were normalized to the signals in GSCs; for the *nos-Gal4ΔVP16; bam-Gal80* combination, all quantifications were also normalized to the signals in GSCs; for *bam-Gal4* by itself, all quantifications were normalized to the signals in the 8-cell SGs (Fig. S1e).

S-Phase colocalization imaging and analysis. To visualize potentially differential histone incorporation during S-phase, we applied a clearance buffer which effectively removes nucleoplasmic protein as previously described (*7, 8*). Briefly, the clearance buffer is prepared by mixing 989µls of the clearance buffer stock solution (8.4 mM HEPES, 100 mM NaCl, 3 mM MgCl, 1 mM EGTA, 300 mM Sucrose, 2% Triton X-1000, and 2% BSA in ddH_2O) with 1 µl

bioRxiv preprint doi: https://doi.org/10.1101/2024.04.26.591383; this version posted April 29, 2024. The copyright holder for this preprint (which was not certified by peer review) is the author/funder, who has granted bioRxiv a license to display the preprint in perpetuity. It is made available under aCC-BY 4.0 International license.

DTT and 10 µl protease inhibitor (100x Leupeptin). After dissection, tissue samples were incubated in 10 µM EdU (Invitrogen Click-iT EdU Imaging Kit, catalog # C10340) for 15 minutes in Schneider's media at room temperature. At the end of the 15 minutes, the Schneider's media were drained and the clearance buffer was added for two minutes at 4°C in darkness. Samples were then fixed in 4% PFA, washed with 1xPBST, and then blocked in 3% BSA for 30 minutes. For robust signals, both the old H3-EGFP and new H3-mCherry were immunostained with antibodies (e.g., anti-EGFP and anti-mCherry) using standard procedures. The CLICK reaction was performed according to manufacturer's instructions to label EdU. The DNA dye Hoechst was also added at this step.

Images were acquired on the Zeiss LSM800 using Airyscan mode on a 63x oil immersion objective. All samples were imaged using the identical settings. GSCs were identified by their proximity to the hub region. When 4-cell stage SGs were used, only one SG per cyst was analyzed to represent one data point. All images were analyzed using FIJI software. The Pearson score was recorded using the Coloc2 plugin for each nucleus, which was cropped to include just the nucleus as much as possible as delineated by the Hoechst signals. For each batch of images, the average measurement of the control GSCs was set to 1 and the other treatments are normalized to control GSCs, in order to avoid batch variability. The resulting values are then used to calculate mean and standard error of the mean (Mean± SEM).

Inhibitor treatment and analysis. For S-phase colocalization experiments using the inhibitor, flies were heat shocked as described above and left at 29°C to recover for 14 hours. Testes were then dissected and placed in incubation media for four hours, resulting in 18 total hours of post-

bioRxiv preprint doi: https://doi.org/10.1101/2024.04.26.591383; this version posted April 29, 2024. The copyright holder for this preprint (which was not certified by peer review) is the author/funder, who has granted bioRxiv a license to display the preprint in perpetuity. It is made available under aCC-BY 4.0 International license.

heat shock recovery. After incubation with the inhibitor at the designated concentrations, these tissues were processed for S-phase colocalization analysis as described above.

Polα180 inhibitor (MedChemExpress Cat# HY-147812), a derivative of the classical inhibitor adarotene, was prepared in DMSO as stock and stored at -20°C (for short term) and -80°C (for long term) according to manufacturer's instructions. Drug incubation was performed on testes in "live cell media" containing Schneider's insect medium with 200 µg/ml insulin, 15% FBS by volume, and 0.6x pen/strep (*9*). Prior to experiments, incubation media was prepared by diluting inhibitor solution (or DMSO vehicle) to the proper concentration in live cell media. Testes were dissected and placed in 100µl of incubation media as quickly as possible following dissection. Incubated testes were left in open tubes in darkness at room temperature (RT) for four hours. Because four hours are longer than the standard S-phase of the early male germline (*10-14*), all S-phase cells at the end of the incubation should have been exposed to the inhibitor for the entirety of their current S-phase.

For EdU incorporation, 20µM EdU was added to the incubation media for the last 15 minutes of the drug incubation before tissue fixation. Only cells in early- to mid-S-phase were used for quantifications, as denoted by EdU staining covering all or most of the nucleus. Cells with focal EdU signal, indicative of late S-phase, were excluded to avoid skewing of the data. Germ cells were determined by endogenously tagged Vasa-mApple signals. Following imaging, EdU incorporation was quantified by measuring the mean EdU signal intensity in EdU-positive germline nuclei and subtracting the background measured from the nearby EdU-negative cells. When a cyst was considered, only one nucleus from each cyst was measured as one data point. Data shown in Figure S2b were based on all early-stage germ cells, as no significant difference

bioRxiv preprint doi: https://doi.org/10.1101/2024.04.26.591383; this version posted April 29, 2024. The copyright holder for this preprint (which was not certified by peer review) is the author/funder, who has granted bioRxiv a license to display the preprint in perpetuity. It is made available under aCC-BY 4.0 International license.

of EdU incorporation was detected among GSCs, GBs, and SGs from the same sample (data not shown).

Generation of chromatin fibers from the *Drosophila* male germline. Chromatin fibers were prepared as previously described (*4*, *15*). Briefly, after adding EdU to the testis samples and incubating for 15 minutes, lysis buffer was added (100 mM NaCl, 25 mM Tris-base, 0.2% Joy detergent, pH=10). The testis tip was then micro-dissected on the slide and the rest of the testis was removed. Cells were allowed to fully lyse for approximately 5 minutes and then a Sucrose/Formalin (1M sucrose; 10% formaldehyde) solution was added and left for 2-minute to incubate, before a cover slip was gently placed on the top. The slide was then transferred to liquid nitrogen for two minutes before the cover slip was removed. The slide was then transferred to 95% EtOH for 10 min at -20°C in a freezer. Afterwards, the slide was fixed in 1% PFA for 1 minute. Samples were washed 3× in a Coplin jar with 1×PBST followed by blocking the sample with 3% BSA in 1×PBST for 30 minutes. Primary antibodies were then added for overnight incubation in a humidity chamber at 4°C. To assess histone asymmetry, anti-PCNA, anti-H3K27me3, and anti-GFP primary antibodies were added to chromatin fibers from the testes from the males with the following genotypes: each of the drivers (*nos-Gal4* itself, *nos-Gal4ΔVP16; bam-Gal80* combination, or *bam-Gal4* itself) crossed with *UASp-FRT-H3-EGFP-FRT-H3-mcherry* without *hs-flp*. For *cdc45-mCherry; DNA Polymerase-HA* fibers, mCherry and HA primary antibodies were used. After the incubation with the primary antibodies, the slides are washed in a coplin jar with 1×PBS. Then the secondary antibodies were added and incubated for two hours at room temperature in a humidity chamber. The click chemistry was performed to label EdU following the manufacturer's instruction. When DNA needs to be labeled, Hoechst is

bioRxiv preprint doi: https://doi.org/10.1101/2024.04.26.591383; this version posted April 29, 2024. The copyright holder for this preprint (which was not certified by peer review) is the author/funder, who has granted bioRxiv a license to display the preprint in perpetuity. It is made available under aCC-BY 4.0 International license.

included at 1:1,000 to stain the samples. Additionally, for samples that need DNA labeling, ProLong™ Gold Antifade Mountant with DNA Stain DAPI (Thermo Fisher catalog # P36931) was used. For samples that do not need DNA labeling, ProLong Diamond mounting media without DAPI (Thermo Fisher catalog# P36961) was used.

Sequential labeling using EdU and BrdU analogs on DNA fibers. After sample dissection, 10 μM EdU was added for a 10-minute incorporation, followed by washing out EdU. BrdU was subsequently added for another 10 minutes. After this sequential labeling, DNA fibers were prepared using the same procedure as described above for chromatin fibers, with the exception of using a different lysis buffer to strip proteins from the DNA (200 mM Tris–HCl, pH 7.5, 50 mM EDTA, 0.5% SDS). The fibers were then treated with 1M HCl for 30 minutes at room temperature to expose the incorporated BrdU. After washing with 1×PBST, BrdU antibodies were added for incubation overnight at 4°C in a humidity chamber. Secondaries antibodies against the BrdU primary antibodies were then added for two hours at room temperature in a humidity chamber. The click reaction to recognize EdU was performed subsequently along with Hoechst incubation at 1:1000. Samples were then mounted in ProLong Diamond mounting media with DAPI. The EdU-positive DNA fibers representing regions that undergo DNA replication during EdU pulse (and thus have EdU on at least one side) were used for subsequent analyses as shown in Figure 5b.

Identifying and imaging replicative DNA fibers and chromatin fibers. All DNA fibers and chromatin fibers in this study were imaged with the Airyscan mode on a Zeiss LSM800 using a

bioRxiv preprint doi: https://doi.org/10.1101/2024.04.26.591383; this version posted April 29, 2024. The copyright holder for this preprint (which was not certified by peer review) is the author/funder, who has granted bioRxiv a license to display the preprint in perpetuity. It is made available under aCC-BY 4.0 International license.

63× oil immersion lens. Germline-derived chromatin fibers were identified using the H3-EGFP signal expressed with different germ cell-specific drivers or driver combination. Replicative regions were identified by both PCNA and EdU signals, or the presence of Cdc45, DNA Polymerase, and EdU. Fibers regions with detectable separation between sister chromatids were imaged and analyzed. Quality controls to select appropriate chromatin fiber regions for further analyses included fiber length, shape, and the molecular specificity of signals. For example, for quantifying old histone-enriched H3K27me3 with strandedness information, the EdU labeled fibers positive with PCNA, H3-EGFP and H3K27me3 signals were used. For analyzing the Cdc45 signals with DNA polymerases, fibers with EdU-labeling regions, clear Cdc45 and anti-HA signals were used.

For sequential EdU and BrdU labeled DNA fibers, two patterns were imaged and quantified at DNA regions that replicate during the EdU pulse (thus incorporating EdU on at least one side of the duplicated sister chromatids): First, regions with clear sister chromatid separation with Hoechst and EdU signals but no discernable BrdU signal. Second, regions with clear sister chromatid separation with clear Hoechst, EdU, and BrdU signals. For detailed description of the analyses of sister chromatids using chromatin fibers, refer to (*4, 15*).

Quantification of DNA fibers and chromatin fibers. All images were analyzed using FIJI software. To quantify the asymmetry between sister chromatids, line plots were drawn on both strands, using the PCNA-enriched side to denote the lagging strand. Most fibers have relatively short separable regions (≤ 2μm), for which the entire fiber was used for quantification. For fibers with longer separable regions (> 2 μm), they were divided into 2μm-long non-overlapping segments along the length of the chromatin fiber and each of them was used for analyses. The

bioRxiv preprint doi: https://doi.org/10.1101/2024.04.26.591383; this version posted April 29, 2024. The copyright holder for this preprint (which was not certified by peer review) is the author/funder, who has granted bioRxiv a license to display the preprint in perpetuity. It is made available under aCC-BY 4.0 International license.

region with no overlap with any of the chromatin fibers was used as background signal for subtraction from the measured signals from both strands. The ratio of signals = $\log_2$ (leading strand signal − background signal) / (lagging strand signal − background signal).

For the sequential EdU- and BrdU-labeled DNA fibers, there is no strandedness indicator such as PCNA. As such, the strand with higher BrdU signals was used as the reference strand, allowing EdU signal to be independently measured, which could be on the same or the opposite strand. All quantifications were performed similar to the chromatin fibers, with the ratio of signals = $\log_2$ (BrdU-enriched strand signal − background signal) / (BrdU-depleted strand signal − background signal).

For the Cdc45- and DNA Polymerase-labeled fibers, the distance between Cdc45 signal and the HA signal (labeling either Polα or Polε) was quantified from the center of the Cdc45 focus to the nearest HA signal.

A quantitative assay for chromosomal condensation state. We used an area-based method to monitor the chromosomal condensation state as previously described (*8*), using a dual-color histone transgene *UASp-FRT-histone-EGFP -FRT-histone-mCherry*. A maximum intensity projection was generated for old H3- (EGFP) and new H3- (mCherry) enriched areas. The intensity of each pixel was determined and scaled individually, setting the minimum intensity to 0 and the maximum to 65,535 (a 16-bit range). We monitored the pixels across the image with a threshold of 35% of the maximum intensity. Condensation kinetic profiles were generated to compare old H3- *versus* new H3-enriched regions by calculating the percentage of pixels above the threshold (the condensation parameter). Relative compaction index was measured and plotted

bioRxiv preprint doi: https://doi.org/10.1101/2024.04.26.591383; this version posted April 29, 2024. The copyright holder for this preprint (which was not certified by peer review) is the author/funder, who has granted bioRxiv a license to display the preprint in perpetuity. It is made available under aCC-BY 4.0 International license.

by taking a ratio of the percentage of pixels of the new H3-enriched to the old H3-enriched regions as described previously (*8*).

Statistics and reproducibility. For all comparisons between two groups, Mann-Whitney tests were used unless otherwise noted. For one-group datasets, one sample *t*-test was used with a null hypothesis that the data is symmetrically distributed (e.g., ratio= 1 for datasets without logarithmic transformation, $\log_2$= 0 for logarithmically transformed data).

Details for Figure 1b

Endogenously expressed Polα-HA levels are significantly depleted in GSCs relative to SGs according to the Mann-Whitney test with a P-value < 10^{-4} (****).

Endogenously expressed Polδ-HA levels are significantly depleted in GSCs relative to SGs according to the Mann-Whitney test with a P-value < 10^{-4} (****).

Endogenously expressed Polε-HA levels are not significantly different between GSCs and SGs according to the Mann-Whitney test with a P-value > 0.05 (= 0.0806, ns).

Endogenously expressed Cdc45-HA levels are not significantly different between GSCs and SGs according to the Mann-Whitney test with a P-value > 0.05 (= 0.8469, ns).

Endogenously expressed Ctf4-EGFP levels are not significantly different between GSCs and SGs according to the Mann-Whitney test with a P-value > 0.05 (= 0.8595, ns).

RPA driven under its own promoter (*rpa>rpa-EGFP*) levels are significantly enriched in GSCs relative to SGs according to the Mann-Whitney test with a P-value < 10^{-4} (****).

bioRxiv preprint doi: https://doi.org/10.1101/2024.04.26.591383; this version posted April 29, 2024. The copyright holder for this preprint (which was not certified by peer review) is the author/funder, who has granted bioRxiv a license to display the preprint in perpetuity. It is made available under aCC-BY 4.0 International license.

Details for Fig. 3f and Fig. S3c

nos-Gal4ΔVP16; bam-Gal80>H3-EGFP (GSC-enriched) chromatin fibers exhibited significantly higher levels of H3K27me3 asymmetry relative to *nanos-Gal4>H3-EGFP* (total early germline) chromatin fibers according to the Mann-Whitney test with a *P*-value < 0.05 (= 0.0250, *).

bam-Gal4>H3-EGFP (SG) chromatin fibers exhibited significantly lower levels of H3K27me3 asymmetry relative to *nanos-Gal4>H3-EGFP* (total early germline) chromatin fibers according to the Mann-Whitney test with a *P*-value < 0.05 (= 0.0296, *).

The *in silico* combination of *nos-Gal4ΔVP16; bam-Gal80>H3-EGFP* (GSC-enriched) and *bam-Gal4>H3-EGFP* (SG) chromatin fibers was not statistically different from the *nanos-Gal4>H3-EGFP* (total early germline) chromatin fibers according to the Mann-Whitney test with a *P*-value > 0.05 (= 0.7458, ns).

nos-Gal4>H3-EGFP; *polα50*$^{+/-}$ chromatin fibers exhibited significantly higher levels of H3K27me3 asymmetry relative to *nanos-Gal4>H3-EGFP* chromatin fibers according to the Mann-Whitney test with a *P*-value < 0.05 (= 0.0256, *).

bam-Gal4>H3-EGFP; *polα50*$^{+/-}$ chromatin fibers exhibited significantly higher levels of H3K27me3 asymmetry relative to *bam-Gal4>H3-EGFP* chromatin fibers according to the Mann-Whitney test with a *P*-value < 0.01 (= 0.0075, **).

bioRxiv preprint doi: https://doi.org/10.1101/2024.04.26.591383; this version posted April 29, 2024. The copyright holder for this preprint (which was not certified by peer review) is the author/funder, who has granted bioRxiv a license to display the preprint in perpetuity. It is made available under aCC-BY 4.0 International license.

nos-Gal4ΔVP16; bam-Gal80>H3-EGFP; *polα50*$^{+/-}$ chromatin fibers were not statistically different from the *nos-Gal4ΔVP16; bam-Gal80>H3-EGFP* chromatin fibers according to the Mann-Whitney test with a *P*-value > 0.05 (= 0.8039, ns).

Details for Fig. S3d

nos-Gal4>H3-EGFP; *polα180*$^{+/-}$ chromatin fibers exhibited significantly higher levels of H3K27me3 asymmetry relative to *nanos-Gal4>H3-EGFP* chromatin fibers according to the Mann-Whitney test with a *P*-value < 0.01 (= 0.0047, **).

nos-Gal4>H3-EGFP; rpa70-HA chromatin fibers exhibited significantly higher levels of H3K27me3 asymmetry relative to *nanos-Gal4>H3-EGFP* chromatin fibers according to the Mann-Whitney test with a *P*-value < 0.05 (= 0.0209, *).

Details for Fig. S4b

For DNA fibers with both EdU and BrdU signals, EdU and BrdU exhibit significantly different distribution according to the Mann-Whitney test with a *P*-value < 10^{-4} (****).

For DNA fibers with both EdU and BrdU signals, BrdU is significantly asymmetrically localized by a one sample t-test with a null hypothesis of $\log_2 = 0$ and a *P*-value < 10^{-4} (****).

For DNA fibers with both EdU and BrdU signals, EdU is significantly asymmetrically localized by a one sample t-test with a null hypothesis of $\log_2 = 0$ and a *P*-value < 10^{-4} (****).

bioRxiv preprint doi: https://doi.org/10.1101/2024.04.26.591383; this version posted April 29, 2024. The copyright holder for this preprint (which was not certified by peer review) is the author/funder, who has granted bioRxiv a license to display the preprint in perpetuity. It is made available under aCC-BY 4.0 International license.

Details for Figure 5g

The chromatin fibers co-labeled with Cdc45-mCherry and Polα-HA exhibit significantly longer distances between mCherry focus and HA signal relative to the chromatin fibers co-labeled with Cdc45-mCherry and Polε-HA by the Mann-Whitney test with a *P*-value < 10^{-4} (****).

Details for Fig. S4d

In *nos-Gal4>H3-EGFP* chromatin fibers, H3K27me3 is significantly asymmetrically localized by a one sample t-test with a null hypothesis of $\log_2 = 0$ and a *P*-value < 10^{-4} (****).

In *nos-Gal4>H3-EGFP* chromatin fibers, PCNA was significantly asymmetrically localized by a one sample t-test with a null hypothesis of $\log_2 = 0$ and a *P*-value < 10^{-4} (****).

In *nos-Gal4>H3-EGFP* chromatin fibers, EdU was not significantly asymmetrically localized by a one sample t-test with a null hypothesis of $\log_2 = 0$ and a *P*-value > 0.05 (= 0.1028, ns).

bioRxiv preprint doi: https://doi.org/10.1101/2024.04.26.591383; this version posted April 29, 2024. The copyright holder for this preprint (which was not certified by peer review) is the author/funder, who has granted bioRxiv a license to display the preprint in perpetuity. It is made available under aCC-BY 4.0 International license.

Supplemental Figures and Figure Legends:

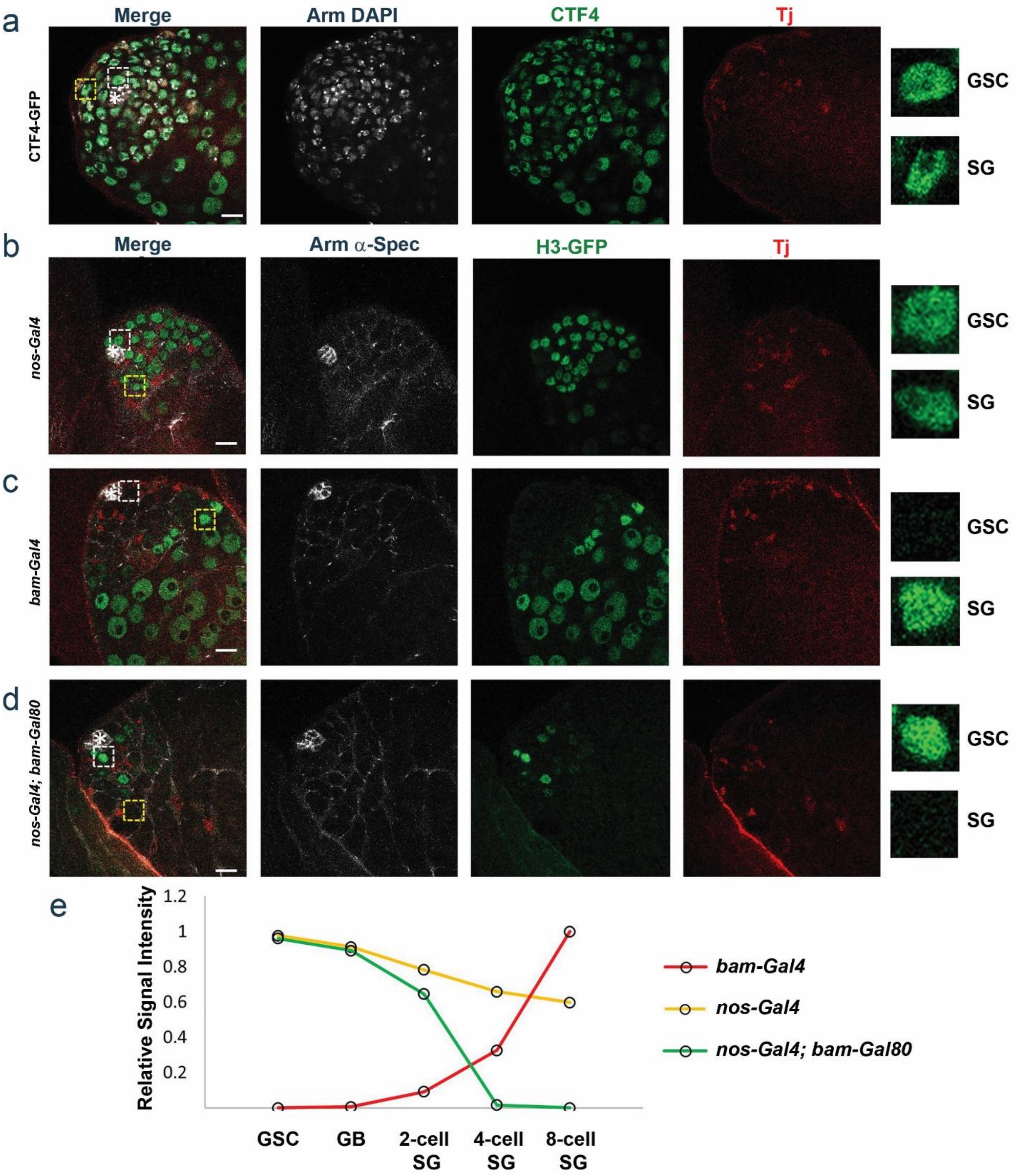

Figure S1: Expression pattern of a replication machinery component CTF4 and distinct expression patterns of an H3-EGFP reporter by different drivers in the *Drosophila* male germline. (**a**) Image of endogenous CTF4-GFP using knock-in strategy (Materials and

bioRxiv preprint doi: https://doi.org/10.1101/2024.04.26.591383; this version posted April 29, 2024. The copyright holder for this preprint (which was not certified by peer review) is the author/funder, who has granted bioRxiv a license to display the preprint in perpetuity. It is made available under aCC-BY 4.0 International license.

Methods): DAPI (white), Arm (white, a marker for hub cells), CTF4-GFP (green), and the somatic marker Traffic Jam (Tj, red). (**b-d**) Images of: (**b**) *nanos-Gal4> UAS-H3-EGFP*, (**c**) *bam-Gal4> UAS-H3-EGFP*, and (**d**) *nos-Gal4ΔVP16; bam-Gal80> UAS-H3-EGFP*, Arm (white), H3-EGFP (green), and the somatic marker Tj (red). (**e**) Quantification of the relative expression levels of H3-EGFP using each corresponding driver (n=3 for each genotyped testes, see Table S2 for details). Asterisk: hub. Scale bar: 10 μm.

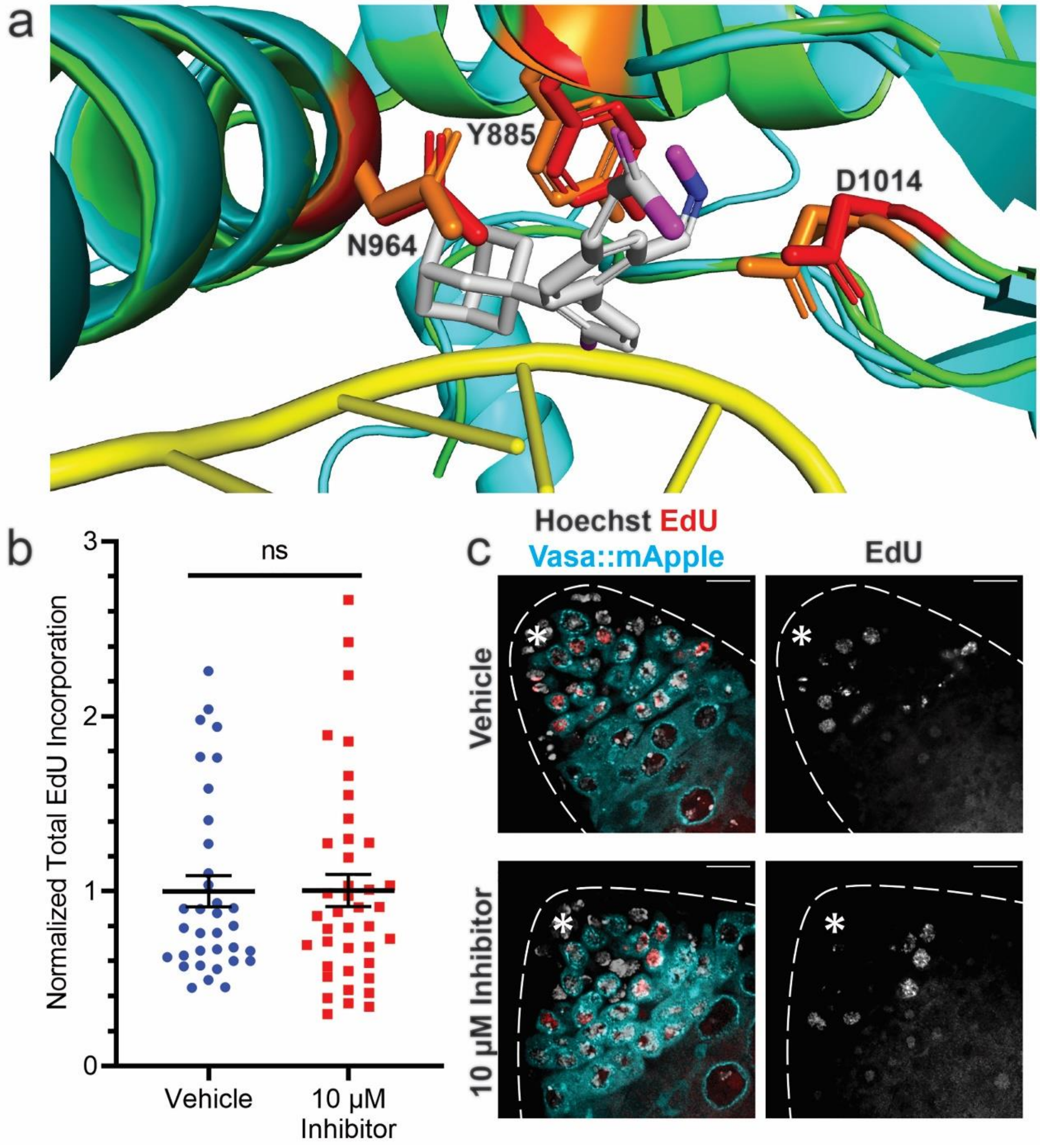

bioRxiv preprint doi: https://doi.org/10.1101/2024.04.26.591383; this version posted April 29, 2024. The copyright holder for this preprint (which was not certified by peer review) is the author/funder, who has granted bioRxiv a license to display the preprint in perpetuity. It is made available under aCC-BY 4.0 International license.

Figure S2: Low concentration of Polα180 (or PolA1) inhibitor partially inhibits Polα while permitting DNA replication. (**a**) PyMOL structural alignment of human DNA Polα (green) with DNA (yellow) (structure: PDB 5IUD) and the AlphaFold prediction of *Drosophila* Polα180 (cyan). The Polα180 inhibitor (carbon: white, oxygen: magenta, nitrogen: blue) is shown at the binding location predicted by (*16*). The Polα180 residues predicted to interact with the inhibitor are conserved between mammals (red) and *Drosophila* (orange). (**b**) Quantification of total EdU incorporation in early-stage germ cells, treated with vehicle or Polα180 inhibitor for four hours, normalized to the mean of vehicle-treated cells. Vehicle treated cells (n=34), 10μM inhibitor treated cells (n=40). Median with first and third quartile shown. Student's t-test, ns: not significant. See Table S5 for details. (**c**) Representative images of testes treated with vehicle or Polα180 inhibitor. In merged images: Hoechst (white), endogenous Vasa-mApple (cyan), EdU (red). Asterisk: hub. Scale bars: 10 μm.

bioRxiv preprint doi: https://doi.org/10.1101/2024.04.26.591383; this version posted April 29, 2024. The copyright holder for this preprint (which was not certified by peer review) is the author/funder, who has granted bioRxiv a license to display the preprint in perpetuity. It is made available under aCC-BY 4.0 International license.

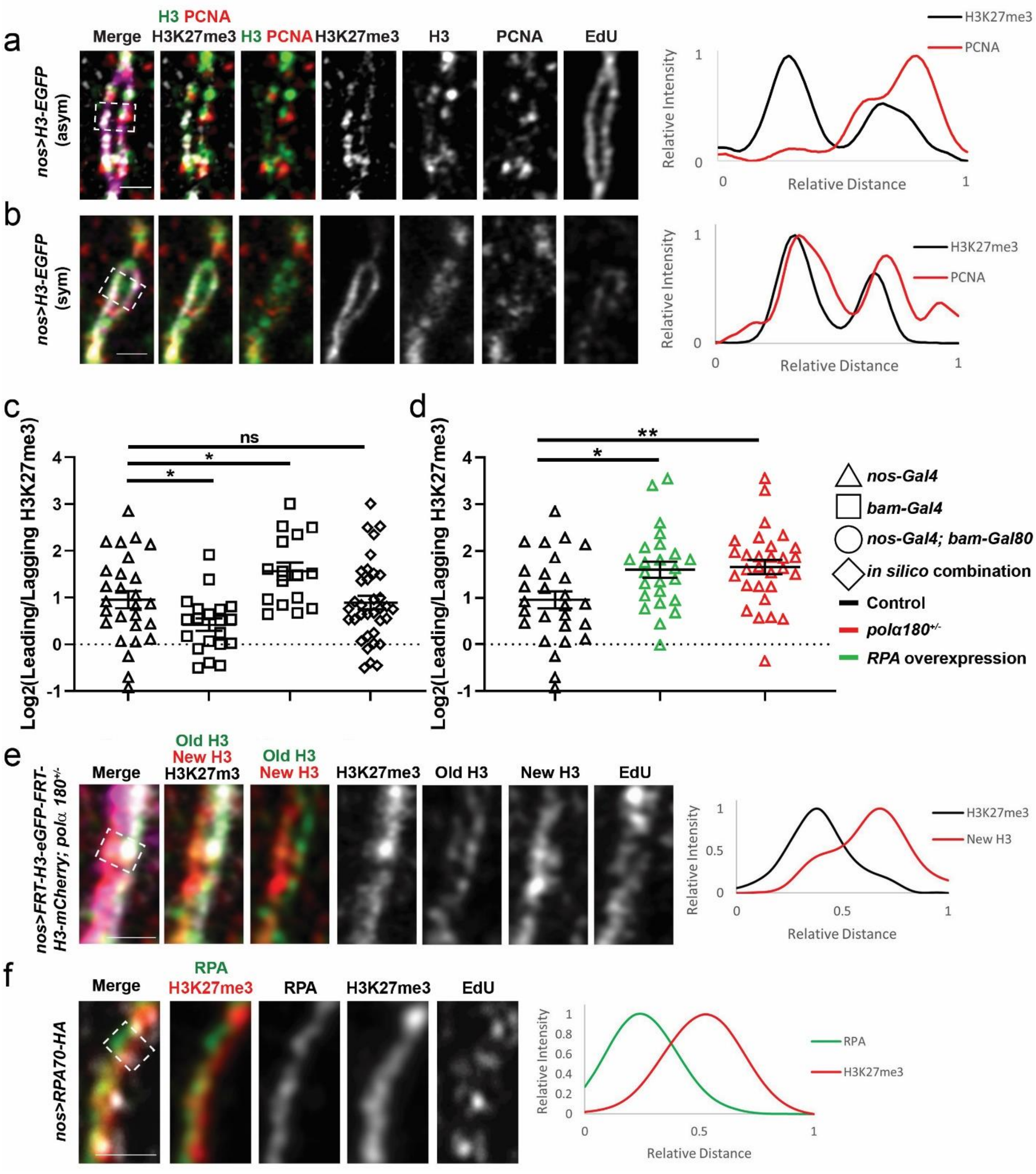

Figure S3: Reducing Polα or enhancing RPA levels increase old histone-enriched H3K27me3 asymmetries at the replication fork. (**a**) An Airyscan image of representative asymmetric *nanos-Gal4>H3-EGFP* chromatin fiber. (**b**) An Airyscan image of representative

bioRxiv preprint doi: https://doi.org/10.1101/2024.04.26.591383; this version posted April 29, 2024. The copyright holder for this preprint (which was not certified by peer review) is the author/funder, who has granted bioRxiv a license to display the preprint in perpetuity. It is made available under aCC-BY 4.0 International license.

symmetric *nanos-Gal4>H3-EGFP* chromatin fiber. In merged images (**a**-**b**): H3K27me3 (white), H3-EGFP (green), PCNA (red), and EdU (magenta). Images in (**a**-**b**) are also accompanied by line plots showing the spatial distribution of H3K27me3 and PCNA signals from the indicated regions, respectively (white dotted outlined box). (**c**) Quantification of H3K27me3 asymmetry using chromatin fibers with *nanos-Gal4>H3-EGFP*, *bam-Gal4>H3-EGFP*, *nos-Gal4ΔVP16*; *bam-Gal80>H3-EGFP*, and an *in silico* combination of *bam-Gal4>H3-EGFP* and *nos-Gal4ΔVP16*; *bam-Gal80>H3-EGFP* in $\log_2$ scale: *nanos-Gal4>H3-EGFP*= 0.95± 0.18 (n=27), *bam-Gal4>H3-EGFP*= 0.42± 0.14 (n=20), *nos-Gal4ΔVP16*; *bam-Gal80>H3-EGFP*= 1.56± 0.19 (n=16), *in silico* combination of *bam-Gal4>H3-EGFP* and *nos-Gal4ΔVP16*; *bam-Gal80>H3-EGFP*= 0.89± 0.15 (n=36). See Table S6 for details. (**d**) Quantification of additional replication protein manipulations using a *nanos-Gal4* driven overexpression of *UAS-rpa70-HA* transgene and a P-element insertion allele of another Polα subunit gene (*polα180*) at a heterozygous background (*polα180*$^{+/-}$) in $\log_2$ scale: *nanos-Gal4>rpa70-HA*= 1.60± 0.17 (n=24), *nanos-Gal4>H3-EGFP; polα180*$^{+/-}$= 1.66± 0.15 (n=29). See Table S7 for details. (**e**) Airyscan image of *hs-flp*; *nanos-Gal4>FRT-H3-EGFP-FRT-H3-mCherry*; *polα180*$^{+/-}$: H3K27me3 (white), old H3 (green), new H3 (red), and EdU (magenta) in the merged image. (**f**) Airyscan image of *nanos-Gal4>rpa70-HA* chromatin fiber: EdU (white), RPA (green), and H3K27me3 (red) in the merged image. Images in (**e**-**f**) are also accompanied by line plots showing the spatial distribution of H3K27me3 and new H3 signals in (**e**), as well as H3K27me3 and RPA signals in (**f**) from the indicated regions, respectively (white dotted outlined box). Scale bar: 1 μm. All ratios: Mean± SEM. All statistics: Mann-Whitney test, **: $P< 0.01$, *: $P< 0.05$, ns: not significant.

bioRxiv preprint doi: https://doi.org/10.1101/2024.04.26.591383; this version posted April 29, 2024. The copyright holder for this preprint (which was not certified by peer review) is the author/funder, who has granted bioRxiv a license to display the preprint in perpetuity. It is made available under aCC-BY 4.0 International license.

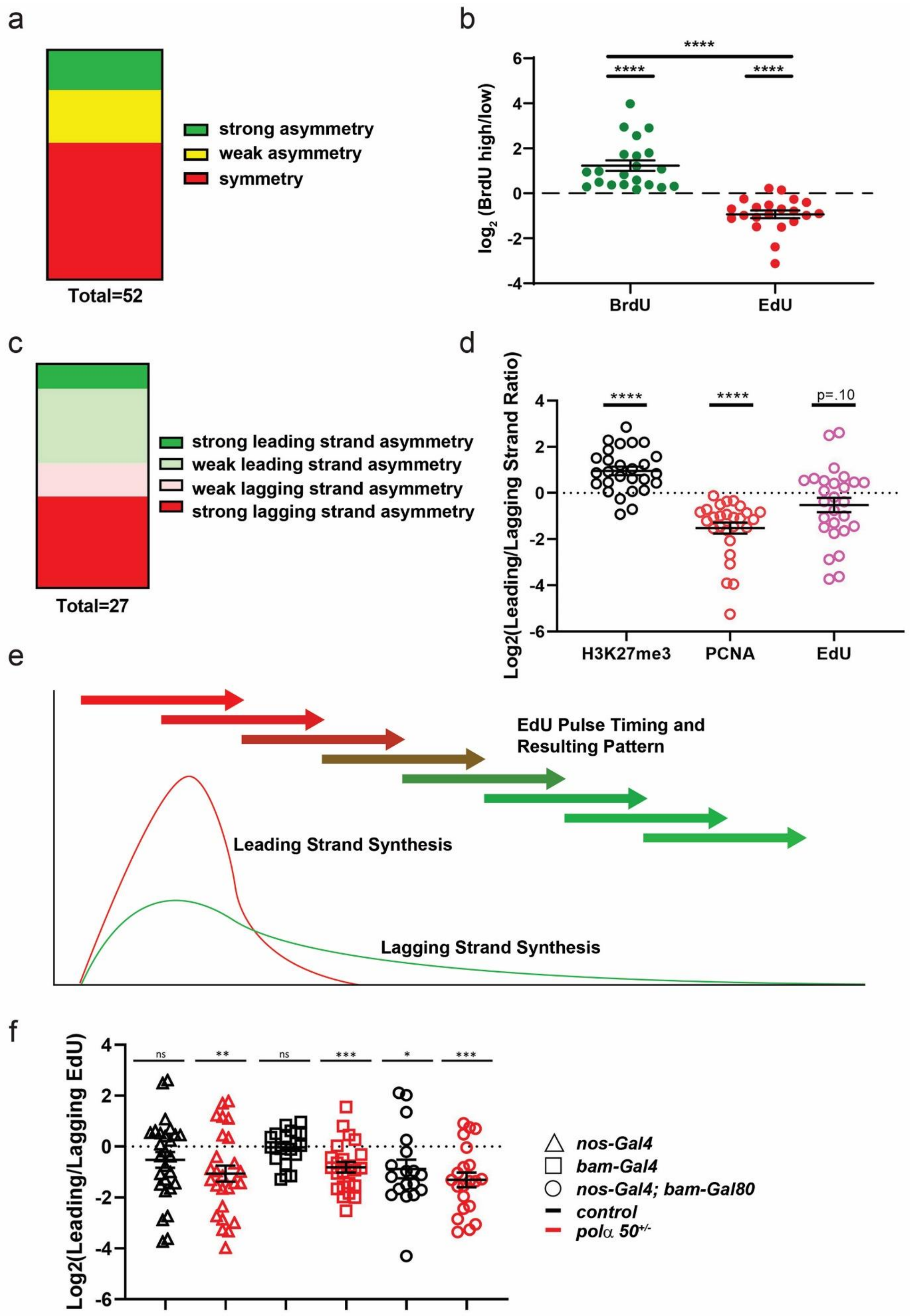

bioRxiv preprint doi: https://doi.org/10.1101/2024.04.26.591383; this version posted April 29, 2024. The copyright holder for this preprint (which was not certified by peer review) is the author/funder, who has granted bioRxiv a license to display the preprint in perpetuity. It is made available under aCC-BY 4.0 International license.

Figure S4: Visualization and quantification of delayed lagging strand synthesis. (**a**) Assessment of observed patterns on DNA fibers, wherein symmetric fibers refer to fibers with EdU on both strands, weak asymmetric fibers have both EdU and BrdU but less than a 2-fold asymmetry on both strands, while strong asymmetric fibers have a greater than 2-fold difference for at least one of the signals (i.e., either EdU or BrdU). The distribution of the three categories are as follows: 60% symmetric, 23% weak asymmetric, 17% strong asymmetric. See Table S9 for details. (**b**) Log_2-scale 1D quantification of EdU and BrdU from the DNA fibers with both signals, where the positive side is the strand with higher BrdU and the negative side is the strand with higher EdU: log_2BrdU= 1.23± 0.23 (n=21), log_2EdU= -0.93± 0.17 (n= 21). ****: $P< 10^{-4}$, Mann-Whitney test for the comparison between two groups, one tailed t-test with a null hypothesis of log_2= 0 (symmetric pattern). See Table S10 for details. (**c**) Assessment of EdU asymmetries wherein ≥ 2-fold are considered strong asymmetry, < 2-fold are considered weak asymmetry. The distribution of the three categories are as follows: 11% strong asymmetry toward the leading strand, 33% weak asymmetry toward the leading strand, 15% weak asymmetry toward the lagging strand, 41% strong asymmetry toward the lagging strand. See Table S13 for details. (**d**) Quantification of H3K27me3, PCNA, and EdU asymmetry from *nos-Gal4>H3-EGFP* labeled chromatin fibers using log_2 scale: log_2H3K27me3= 0.95± 0.18 (n=27), log_2PCNA= -1.52± 0.24 (n=27), log_2EdU= -0.52± 0.31 (n=27). ****: $P< 10^{-4}$, one tailed t-test with a null hypothesis of log_2= 0 (symmetric pattern). See Table S14 for details. (**e**) A model of replication patterns that could explain the observed EdU patterns. (**f**) Quantification of EdU distribution on chromatin fibers labeled with H3-EGFP driven by the following drivers with regard to the strandedness using log_2 scale: *nanos-Gal4*= -0.52± 0.31 (n=27, P= 0.103, ns), *nanos-Gal4*; *polα50*$^{+/-}$= -1.06± 0.32 (n=26, $P< 0.01$), *bam-Gal4*= -0.03± 0.14 (n=21, P= 0.817,

bioRxiv preprint doi: https://doi.org/10.1101/2024.04.26.591383; this version posted April 29, 2024. The copyright holder for this preprint (which was not certified by peer review) is the author/funder, who has granted bioRxiv a license to display the preprint in perpetuity. It is made available under aCC-BY 4.0 International license.

ns), *bam-Gal4*; *polα50*$^{+/-}$= -0.081± 0.21 (n=23, *P*< 10^{-3}), *nos-Gal4ΔVP16*; *bam-Gal80*= -0.88± 0.37 (n=18, *P*< 0.05), *nos-Gal4ΔVP16*; *bam-Gal80*; *polα50*$^{+/-}$= -1.31± 0.29 (n=21, *P*< 10^{-3}). All ratios: Mean± SEM, one tailed t-test with a null hypothesis of $\log_2$= 0 (symmetric pattern), ***: *P*< 10^{-3}, **: *P*< 0.01, *: *P*< 0.05, ns: not significant. See Table S11 for details.

Supplemental Tables

Table S1: Raw Data Related to Figure 1b ($\log_2$ scale):

GSC Polα	SG Polα	GSC Polδ	SG Polδ	GSC Polε	SG Polε	GSC Cdc45	SG Cdc45
-1.31257	0.478214	-0.53529	0.025996	-0.05733	0	-0.40234	-0.18844
0.309191	0.179521	-0.22863	0.522421	0.108252	-0.28951	0.118644	-0.34792
-0.37796	0.34155	-0.82902	-0.28951	0.192645	-0.11704	-0.18844	-0.10692
-0.58645	-0.30091	-1.13936	-1.02647	0.036995	0.303069	0.166649	0.014646
0.282692	-0.70322	-1.25966	0.368388	-0.1375	0	0.111576	-0.02975
-0.8576	-1.69149	-1.08278	0.172836	-0.33605	0.37707	-0.25716	-0.18844
-1.74014	0.843809	-1.02833	0.115881	-0.4339	0.272372	0.014646	0
-1.29675	-0.41973	-1.78322	-0.35509	0.125531	-0.2007	0.032029	-0.00295
-1.39827	-1.2292	-1.78322	-0.73696	-0.01886	-0.3599	0.166649	-0.32931
-1.09303	0.909612	-1.46129	-0.1375	-0.22239	-0.48543	-0.50619	0.179706
-2.0808	0.249741	-0.35695	0	-0.56635	-0.48543	0.192645	0.099536
-2.41359	-1.2475	-0.61329	0.466568	0.159479	-0.38414	0.057715	0.20547
-2.87359	-0.29352	-1.19826	0	0.347923	-0.28951	0.192645	0.179706
-1.72364	-0.34207	-0.97586	-0.1375	0.036995	-0.03797	-0.31093	-0.0148
-0.10579	-0.23503	-0.82902	-0.2115	-0.24442	-0.4339	-0.20532	0.304153
-0.16098	-0.27503	-1.08278	-0.49596	-0.4088	-0.5119	0.099536	-0.32931
-1.39736	0.492338	-0.46129	-0.06711	-0.46667	-0.65208	-0.50773	0.166649
-0.9895	0.601421		-0.67283	-0.42182	-0.31259	0.166649	0.140178
-0.65638	0.397087		-0.08927	-0.5771	0.036995	0.071791	0.126757
-0.59786	-0.77119		-0.65703	-0.73275	-0.26679	0.113211	0.065614
-1.33687	0.605119		-0.78587		-0.05733	-0.32931	-0.44479
-0.58599	0.530551		-0.2863		0.192645		
-0.87321	0.278698		-0.08092		0.287803		
-1.41299	-1.55611		0		0.318177		
-0.55373	0.58095		0.172836		0.256775		
-0.8475	-0.91912		0.125532		0.303069		

bioRxiv preprint doi: https://doi.org/10.1101/2024.04.26.591383; this version posted April 29, 2024. The copyright holder for this preprint (which was not certified by peer review) is the author/funder, who has granted bioRxiv a license to display the preprint in perpetuity. It is made available under aCC-BY 4.0 International license.

-1.17455	0.525471		0.076621		0		
-0.84107	0.469582		0.327165		-0.17932		
-0.06675	0.312027		0.149378		0.272372		
0.17612	0.67072		-0.10893		-0.03797		
0.604322	1.002294		0.327165		-0.24442		
-0.64542	0.194597		0.558491		-0.1375		
-0.10228	-0.09784		0.172836		-0.45943		
-0.49791	0.424877		-0.1375		-0.1375		
-2.1112	0.425666		0.742202		0.36257		
-1.29082	0.549028		0.862497		0.241008		
-2.74306	-0.42891		0.149378		0.125531		
	-0.68767		0.149378		0.192645		
	-1.09098		0.241008		0.287803		
	0.16498		-0.05344		0.347923		
	-0.26264		-0.1964		0.256775		
	-0.3827		0.7578		0.018616		
	-0.2624		0.77323		0.287803		
	0.338635		0.803603				
	0.196455		0.742202				
	0.017312		0.803603				
	-0.28549		0.59368				
	-1.18792		0.833351				
	0.486708		0.77323				
	0.583987		0.263035				
	-0.38879		-1.45943				
	-0.38339		-1.38904				
	0.96703		-1.02647				
	-1.51239		-1.32193				
	-0.35785		-0.65208				
	0.16457		-0.61143				
	-0.42837		-1.08092				
	-0.91937		-0.92338				
	-0.16938		-0.53343				
	-0.24721		-1.45223				
	-0.32259		0.412413				
	-2.00388		0.847997				
	-0.63923		0.347924				
	-0.93225		0.051531				
	-2.37993		-0.53343				
	-0.04774		-0.10893				
	-1.16026		-0.6939				

bioRxiv preprint doi: https://doi.org/10.1101/2024.04.26.591383; this version posted April 29, 2024. The copyright holder for this preprint (which was not certified by peer review) is the author/funder, who has granted bioRxiv a license to display the preprint in perpetuity. It is made available under aCC-BY 4.0 International license.

	-0.20723		-0.78136				
	-1.70132		0.788496				
	-0.61486		0.803603				
	-0.65722						
	0.211628						
	0.056212						
	-2.66654						
	-0.89585						
	-1.06545						
	-0.15041						
	-0.32111						
	0.264392						
	-0.59622						
	0.09556						
	0.560539						
	0.63656						
	0.314024						
	-0.00704						
	-2.17927						
	-1.23407						
	-0.51216						
	-0.34315						
	-0.6354						
	-0.18619						
	0.56031						
	0.374148						
	0.559079						
	0.300244						
	-0.49019						
	-1.13866						
	0.79303						
	0.440277						
	0.96703						
	0.741985						
	-1.01161						
	-0.22325						
	-0.27899						
	0.875468						
	0.510143						
	0.5178						
	0.713183						

bioRxiv preprint doi: https://doi.org/10.1101/2024.04.26.591383; this version posted April 29, 2024. The copyright holder for this preprint (which was not certified by peer review) is the author/funder, who has granted bioRxiv a license to display the preprint in perpetuity. It is made available under aCC-BY 4.0 International license.

	0.633194						
	0.886434						
	0.439395						
	0.835981						
	0.491163						
	0.432999						
	0.097461						
	0.06878						
	0.095233						
	0.007007						

Raw Data Related to Figure 1b (continued):

GSC Ctf4	SG Ctf4	GSC RPA	SG RPA
0.623142	0.951972	0.71413	0.46296
0.609015	-0.50849	0.148959	0.63969
0.171094	-0.1383	0.511862	-0.05705
-0.35686	-0.74374	0.6915	0.228219
0.066299	0.490909	0.75115	-0.01768
-0.04728	-0.26161	0.508111	0.396194
0.243402	0.802319	0.644909	-0.98263
0.353389	-0.05505	0.410073	-0.63155
0.204584	0.254205	0.951807	-0.71856
0.573534	-0.79276	0.699907	-0.57468
-0.00298	0.196288	0.580045	-0.30973
0.053226	0.364334	0.606322	-2.00897
0.127204	-0.37851	0.762479	-2.80474
0.210356	0.278883	0.529572	-1.82867
0.003056	0.452054	0.494567	-0.90634
-0.30331	-0.29025	0.683544	-0.8102
-0.08452	-0.07451	0.426963	-0.58593
0.275981	0.105654	1.051248	0.162835
0.103751	0.704144	0.686968	0.149163
-0.18246	-0.3556	-0.21195	-0.01045
0.090752	-0.16395	0.174263	0.939597
-0.35899	0.116547	0.451457	-0.16126
-0.01312	0.362708	0.555232	0.094393
0.064503	0.040772		0.021406
-0.1226	-0.06534		-0.68737
-0.39215	-0.35847		-0.74938

bioRxiv preprint doi: https://doi.org/10.1101/2024.04.26.591383; this version posted April 29, 2024. The copyright holder for this preprint (which was not certified by peer review) is the author/funder, who has granted bioRxiv a license to display the preprint in perpetuity. It is made available under aCC-BY 4.0 International license.

0.262724	0.742378		0.174443
-0.01062	-0.31665		0.088719
0.123721	0.370067		-0.58799
-0.19653	-0.4246		-0.45357
0.134335	-0.49635		-0.10598
-0.07488	0.011934		-0.28448
0.118006	-0.29622		0.457047
0.68878	0.201033		0.438428
-0.36554	0.608532		0
-0.24475	-0.42571		0.239108
0.013141	0.241597		0.511733
-0.44443	0.268592		0.605868
-0.32045	-1.04457		0.445787
-0.27927	-0.01203		0.268457
			0.261867
			-0.94206
			0.465529
			-1.45961
			-0.445
			-1.92342
			-0.26846
			-0.78815
			-0.07116
			0.389252
			0.200518
			0.615396
			0.309905
			0.642078
			0.611062
			0.38686
			0.141967

Table S2: Raw Data Related to Figure S1e:

nos-Gal4 relative expression			
	Testis 1	Testis 2	Testis 3
GSC	1	0.930712	1
GB	0.771986	1	0.965367
2-cell SG	0.925594	0.632471	0.789276
4-cell SG	0.641036	0.734639	0.600625

bioRxiv preprint doi: https://doi.org/10.1101/2024.04.26.591383; this version posted April 29, 2024. The copyright holder for this preprint (which was not certified by peer review) is the author/funder, who has granted bioRxiv a license to display the preprint in perpetuity. It is made available under aCC-BY 4.0 International license.

8-cell SG	0.596229	0.620937	0.574162

bam-Gal4 relative expression			
	Testis 1	Testis 2	Testis 3
GSC	0	0	0
GB	0	0.016474	0
2-cell SG	0.186506	0.082372	0.00276
4-cell SG	0.347403	0.194399	0.438473
8-cell SG	1	1	1

nos-Gal4ΔVP16; bam-Gal80 relative expression			
	Testis 1	Testis 2	Testis 3
GSC	1	0.883686	1
GB	0.968259	1	0.709542
2-cell SG	0.968259	0.745044	0.223784
4-cell SG	0	0	0.04642
8-cell SG	0	0	0

Table S3: Raw Data Related to Figure 2c:

WT GSC	WT SG	*polα50*$^{+/-}$ GSC	*polα50*$^{+/-}$ SG
1.05	1.138636	0.986842	1.131579
1.009091	1.077273	1.013158	1.026316
0.940909	1.138636	0.957613	1.026817
0.942308	1.20858	1.052768	0.966263
1.127219	1.326923	0.927336	1.000865
0.927515	1.275148	1.031142	1.031142
1.08284	1.289941	1.031142	1.07872
0.920118	1.087209	1.012857	1.031142
0.994186	1.139535	1.087143	0.966263
1.005814	1.048246	0.944286	1.035467
1	1.039474	0.955714	1.161429
0.881579	1.372807		1.001429
0.995614	1.372807		1.018572
0.995614	1.232456		0.932857
1.004386	1.355263		0.852857
1.083333	1.47807		0.921429
1.039474			

bioRxiv preprint doi: https://doi.org/10.1101/2024.04.26.591383; this version posted April 29, 2024. The copyright holder for this preprint (which was not certified by peer review) is the author/funder, who has granted bioRxiv a license to display the preprint in perpetuity. It is made available under aCC-BY 4.0 International license.

Table S4: Raw Data Related to Figure 2f:

GSC				SG			
Vehicle	2.5µM inhibitor	5.0µM inhibitor	10µM inhibitor	Vehicle	2.5µM inhibitor	5.0µM inhibitor	10µM inhibitor
1.109244	1.092308	1.051546	1.086792	1.092437	0.969231	0.943299	1.207547
0.890756	1.015385	1.128866	1.041509	1.243697	1.184615	0.974227	0.860377
0.981132	1.107692	0.912371	0.739623	1.327731	1	1.14433	1.116981
1.14717	1.092308	1.128866	1.056604	1.142857	1.230769	1.113402	1.056604
0.996226	0.938462	1.020619	0.981132	1.207547	1.107692	1.14433	1.011321
0.875472	1.046154	1.121212	1.116981	1.056604	0.876923	1.221649	1.177358
0.943089	0.984615	0.878788	1.071698	1.177358	0.969231	1.206186	1.086792
1.056911	1.076923	0.863636	1.14433	1.298113	1.138462	0.742424	1.132075
1.082474	1.046154	0.848485	1.06701	1.25283	1.2	1.19697	0.875472
0.881443	1.097938	1.121212	1.097938	1.011321	1.138462	1.272727	0.935849
1.036082	1.113402	1.166667	1.175258	1.298113	1.107692	1.19697	1.221649
1.045455	1.097938	1.030303	1.075758	1.071698	1.237113	0.772727	0.896907
1.075758	1.06701		0.833333	1.298113	1.221649	0.863636	0.909091
0.80303	0.896907		1.015152	1.086792	1.190722	0.939394	0.833333
1.075758			1.136364	1.14717	1.376289	1.287879	1.106061
				1.170732	1.190722	0.863636	0.772727
				1.235772	0.958763	1.227273	1.166667
				1.284553	1.314433	1.060606	
				1.283505	1.036082	1.212121	
				1.268041	1.268041		
				1.051546	0.927835		
				1.237113	1.252577		
				1.237113	1.175258		
				1.082474			
				1.391753			
				1.333333			
				1.333333			
				1.212121			

Table S5: Raw Data Related to Figure S2b:

Vehicle	10µM inhibitor
1.768912232	0.2980559439
1.406431678	0.5703733404
1.979891974	0.7134650875

bioRxiv preprint doi: https://doi.org/10.1101/2024.04.26.591383; this version posted April 29, 2024. The copyright holder for this preprint (which was not certified by peer review) is the author/funder, who has granted bioRxiv a license to display the preprint in perpetuity. It is made available under aCC-BY 4.0 International license.

1.94082861	0.7286883134
1.10248142	1.03228703
2.04038843	0.6820866272
1.763995807	0.5027542097
0.4519752608	0.8589465744
0.554655396	0.974906994
0.6014346335	0.4181676697
0.9006407924	0.3394453165
0.4929981929	0.8810669726
1.036008703	0.5115177982
2.258700195	0.9094346865
0.7607533816	0.5911148828
0.6580655943	0.4353717814
0.932866977	0.360196572
0.5751706881	0.6759155606
0.6235023975	0.5448133858
0.6328533306	1.659652875
0.7897882994	1.007774796
0.6006922576	0.7858743857
0.6686981052	0.3895679274
0.8022236996	1.033776955
0.8981248001	1.550352538
0.4481664552	1.277899902
0.6601635715	1.415550213
0.8761503076	1.299360521
0.9032685344	2.666303025
0.6781361046	2.42499605
1.271230475	0.9075509585
0.5710077592	0.6923755381
0.7617371828	1.892891999
1.588056753	1.192669706
	0.9881308206
	1.856820102
	2.23577449
	0.7922893526
	1.274524202
	0.8001802708

Table S6: Raw Data Related to Figure 3f and Figure S3c:

nos-Gal4	*bam-Gal4*	*nos-Gal4ΔVP16; bam-Gal80*	*in Silico nos-Gal4ΔVP16*; *bam-Gal80* and *bam-Gal4*
0.833755	0.023199	3.012224	0.023199
1.205462	0.002493	1.520095	0.002493
0.067381	0.913725	0.963087	0.913725
2.185072	0.557483	0.845611	0.557483
0.596007	-0.09516	0.674583	-0.09516

bioRxiv preprint doi: https://doi.org/10.1101/2024.04.26.591383; this version posted April 29, 2024. The copyright holder for this preprint (which was not certified by peer review) is the author/funder, who has granted bioRxiv a license to display the preprint in perpetuity. It is made available under aCC-BY 4.0 International license.

1.240573	0.64262	1.358367	0.64262
2.138097	0.534351	2.503381	0.534351
1.509625	-0.39413	1.484098	-0.39413
1.578043	0.243138	0.998465	0.243138
-0.70103	0.177005	0.769789	0.177005
0.720501	0.535789	2.527987	0.535789
0.441195	1.919735	0.647042	1.919735
0.055739	0.067541	2.353069	0.067541
-0.92427	0.509258	2.198496	0.509258
0.628984	0.753873	1.563693	0.753873
0.870491	0.761944	1.608681	0.761944
0.460992	-0.45322		-0.45322
1.861391	0.810568		0.810568
1.048597	-0.50049		-0.50049
0.120114	1.389966		1.389966
0.916928			3.012224
2.28547			1.520095
-0.25156			0.963087
2.853039			0.845611
0.400734			0.674583
2.197903			1.358367
1.424744			2.503381
			1.484098
			0.998465
			0.769789
			2.527987
			0.647042
			2.353069
			1.563693
			1.608681

Raw Data Related to Figure 3f (continued):

nos-Gal4; polα50$^{+/-}$	*bam-Gal4; polα50*$^{+/-}$	*nos-Gal4ΔVP16; bam-Gal80; polα50*$^{+/-}$
1.71324	-0.25949	1.130421
1.680616	0.614043	1.889422
1.085999	1.229249	1.55281
1.620811	0.831694	0.938152
1.004243	1.557561	1.519162
1.134904	1.30331	0.997146

bioRxiv preprint doi: https://doi.org/10.1101/2024.04.26.591383; this version posted April 29, 2024. The copyright holder for this preprint (which was not certified by peer review) is the author/funder, who has granted bioRxiv a license to display the preprint in perpetuity. It is made available under aCC-BY 4.0 International license.

0.538452	1.387124	1.289368
1.377369	0.413369	0.700357
0.209853	2.71423	0.66006
1.277605	0.927196	1.754169
0.165427	0.02529	2.32908
2.37251	0.590838	0.938002
1.188624	1.190953	2.784909
1.429034	1.138515	1.103144
2.772322	1.336803	2.021303
3.005586	2.862607	2.376666
2.465316	0.426856	1.520804
1.284234	0.29905	0.642919
1.706529	1.812977	1.529886
1.52534	3.308225	1.460099
1.517268	-0.05976	1.396838
1.403463	1.058061	1.719726
1.667895		

Table S7: Raw Data Related to Figure S3d:

polα180$^{+/-}$	*nos>rpa*
1.660701	2.601037
2.294621	-0.01006
2.100029	1.819792
1.851209	2.076201
1.870423	1.81073
1.913859	0.444184
1.541163	0.941755
2.06779	1.629409
1.977457	1.043768
0.570688	1.940334
2.604591	1.542164
1.23827	0.883738
0.96408	3.404706
0.582147	1.142323
0.542417	1.307931
1.507558	1.755604
1.631278	2.376969
1.874469	1.397628
3.300395	0.685891

bioRxiv preprint doi: https://doi.org/10.1101/2024.04.26.591383; this version posted April 29, 2024. The copyright holder for this preprint (which was not certified by peer review) is the author/funder, who has granted bioRxiv a license to display the preprint in perpetuity. It is made available under aCC-BY 4.0 International license.

3.562242	2.149329
2.217418	3.55259
1.607232	0.768625
1.243226	1.818162
0.721922	1.345405
1.470142	
1.264972	
-0.35318	
2.33985	
1.881944	

Table S8: Raw Data Related to Figure 4e: Compaction index using log_2 scale in control and *pola50*$^{+/-}$ GSCs and 8-cell SGs.

GSC	SG	*pola50*$^{+/-}$ GSC	*pola50*$^{+/-}$ SG
1.051	0.581	1.868	1.409
1.948	0.210	0.539	1.825
1.070	0.658	1.358	1.233
0.966	0.554	0.814	1.410
1.588	0.507	0.787	1.692
0.959	0.565	0.607	0.842
1.491	0.215	1.330	2.127
1.166	0.129	1.992	2.262
1.859	0.515	0.790	1.478
0.679	0.369	2.284	1.312
1.376	0.370	0.792	0.747
2.800	0.201	0.975	
0.676		1.616	
0.831			
0.557			

Table S9: Related to Figure S4a:

Symmetric (EdU/EdU fiber)	Weak Asymmetry (EdU/BrdU less than 2-fold asymmetry)	Strong Asymmetry (EdU/BrdU with greater than 2-fold asymmetry)
31	12	9

Table S10: Raw Data Related to Figure 5c and Figure S4b:

Log_2(BrdU)	Log_2(EdU)
0.949727	-0.78622

bioRxiv preprint doi: https://doi.org/10.1101/2024.04.26.591383; this version posted April 29, 2024. The copyright holder for this preprint (which was not certified by peer review) is the author/funder, who has granted bioRxiv a license to display the preprint in perpetuity. It is made available under aCC-BY 4.0 International license.

3.979822	-2.37851
2.947978	-3.12338
0.975338	-0.26303
0.494109	-0.52319
1.794681	-0.69707
0.313499	-0.90752
0.264416	0.147226
0.285157	-0.40526
2.560361	-1.06054
0.377185	-0.94404
2.900464	-1.48543
0.385654	-0.24246
1.201369	-1.25831
1.076044	-0.61857
1.662965	-1.5025
1.730393	0.21864
0.171511	-0.98296
0.587198	-0.9783
0.373352	-0.69574
0.830075	-1.11346

Table S11: Log_2 Raw Data Related to Figure 5d and Figure S4f*:

nos-Gal4	*nos-Gal4; polα50$^{+/-}$*	*bam-Gal4*	*bam-Gal4; polα50$^{+/-}$*	*nos-Gal4ΔVP16; bam-Gal80*	*nos-Gal4 ΔVP16; bam-Gal80; polα50$^{+/-}$*
-1.64051	1.706293	0.539826	0.456845	-0.73765	-1.36482
2.612107	-3.25913	0.140349	-1.57106	-0.95258	0.75138
0.451143	0.354129	0.035564	-1.05006	-1.89181	-0.03933
-1.74333	1.783676	-1.15265	-0.75268	-1.1849	0.70204
0.546183	-1.6443	-0.31179	0.033124	2.108614	-1.28129
-2.88245	-0.65438	0.959769	0.805337	-1.90253	-1.04057
-3.72782	-2.72158	-0.0864	-2.00483	-1.708	-0.73152
-1.44057	-3.32288	-0.47096	-2.517	-1.46108	-3.05472
-3.62121	-0.39184	0.849896	-0.83188	-4.2996	-2.8483
-2.73246	-0.87832	-0.03163	-0.31128	-1.11643	-2.33536
0.096365	-0.95884	0.505262	-1.53716	0.266644	-3.35477
0.701662	-1.14961	-0.1108	-0.42537	-0.20308	-1.95236
2.49744	0.442487	0.371136	0.274047	-1.95157	-1.36273
-0.17693	-2.98338	0.388789	-1.43808	1.353261	-1.56238
0.415777	-0.97708	0.515224	-0.99389	-0.96038	0.908932
0.63151	-1.30605	-0.28391	-0.84444	-1.6619	-1.53928
-0.7567	1.10949	0.62232	-0.5031	2.020612	-2.43426
-0.99991	1.181099	-1.15803	-1.97743	-1.48487	-3.2655

bioRxiv preprint doi: https://doi.org/10.1101/2024.04.26.591383; this version posted April 29, 2024. The copyright holder for this preprint (which was not certified by peer review) is the author/funder, who has granted bioRxiv a license to display the preprint in perpetuity. It is made available under aCC-BY 4.0 International license.

-1.29837	1.226325	-0.03333	-1.83919		0.490629
0.530978	-1.62792	-0.69519	-1.66125		-1.31617
-1.07129	-2.34696	-1.28691	-0.6374		-0.80856
-0.36712	-1.43265		-0.91854		
0.237249	-1.42746		1.550994		
-1.4912	-1.52134				
1.076845	-3.97101				
-0.37702	-2.85406				
0.469742					

(*Data are provided in absolute values in Fig.5d and $\log_2$ values in Fig.S4f)

Table S12: Raw Data Related to Figure 5g:

Polα	Polε
0.581	0.531
4.038	0.6
7.409	0.497
1.922	0.368
2.366	1.269
2.908	0.698
3.641	0.822
0.983	0.727
1.577	0.776
3.312	0.316
3.657	0.908
1.293	0.309
2.333	0.223
8.184	0.161
	0.147

Table S13: Related to Figure S4c:

Strong asymmetry toward the leading strand (≥ 2-fold)	Weak asymmetry toward the leading stand (< 2-fold)	Weak asymmetry toward the lagging stand (< 2-fold)	Strong asymmetry toward the lagging strand ((≥ 2-fold)
3	9	4	11

Table S14: Related to Figure S4d:

PCNA	EdU	H3K27me3
-1.05482	-1.64051	0.833755
-1.21579	2.612107	1.205462
-0.3613	0.451143	0.067381

bioRxiv preprint doi: https://doi.org/10.1101/2024.04.26.591383; this version posted April 29, 2024. The copyright holder for this preprint (which was not certified by peer review) is the author/funder, who has granted bioRxiv a license to display the preprint in perpetuity. It is made available under aCC-BY 4.0 International license.

-0.85911	-1.74333	2.185072
-0.7166	0.546183	0.596007
-5.24247	-2.88245	1.240573
-1.49709	-3.72782	2.138097
-0.56181	-1.44057	1.509625
-2.67029	-3.62121	1.578043
-3.0681	-2.73246	-0.70103
-0.49276	0.096365	0.720501
-1.1499	0.701662	0.441195
-0.82622	2.49744	0.055739
-0.12406	-0.17693	-0.92427
-1.09902	0.415777	0.628984
-0.99608	0.63151	0.870491
-1.45258	-0.7567	0.460992
-2.06082	-0.99991	1.861391
-0.93227	-1.29837	1.048597
-1.53805	0.530978	0.120114
-0.83251	-1.07129	0.916928
-3.9494	-0.36712	2.28547
-3.90744	0.237249	-0.25156
-1.45679	-1.4912	2.853039
-0.33855	1.076845	0.400734
-1.61222	-0.37702	2.197903
-1.04414	0.469742	1.424744

bioRxiv preprint doi: https://doi.org/10.1101/2024.04.26.591383; this version posted April 29, 2024. The copyright holder for this preprint (which was not certified by peer review) is the author/funder, who has granted bioRxiv a license to display the preprint in perpetuity. It is made available under aCC-BY 4.0 International license.

Supplemental References:

1. M. Van Doren, A. L. Williamson, R. Lehmann, Regulation of zygotic gene expression in Drosophila primordial germ cells. *Curr Biol* **8**, 243-246 (1998).
2. M. Inaba, M. Buszczak, Y. M. Yamashita, Nanotubes mediate niche-stem-cell signalling in the Drosophila testis. *Nature* **523**, 329-332 (2015).
3. D. Chen, D. M. McKearin, A discrete transcriptional silencer in the bam gene determines asymmetric division of the Drosophila germline stem cell. *Development* **130**, 1159-1170 (2003).
4. M. Wooten *et al.*, Asymmetric histone inheritance via strand-specific incorporation and biased replication fork movement. *Nat Struct Mol Biol* **26**, 732-743 (2019).
5. S. A. Blythe, E. F. Wieschaus, Zygotic genome activation triggers the DNA replication checkpoint at the midblastula transition. *Cell* **160**, 1169-1181 (2015).
6. V. Tran, C. Lim, J. Xie, X. Chen, Asymmetric division of Drosophila male germline stem cell shows asymmetric histone distribution. *Science* **338**, 679-682 (2012).
7. T. D. Carroll, I. P. Newton, Y. Chen, J. J. Blow, I. Nathke, Lgr5(+) intestinal stem cells reside in an unlicensed G1 phase. *J Cell Biol* **217**, 1667-1685 (2018).
8. R. Ranjan *et al.*, Differential condensation of sister chromatids acts with Cdc6 to ensure asynchronous S-phase entry in Drosophila male germline stem cell lineage. *Dev Cell* **57**, 1102-1118 e1107 (2022).
9. R. Ranjan, J. Snedeker, X. Chen, Asymmetric Centromeres Differentially Coordinate with Mitotic Machinery to Ensure Biased Sister Chromatid Segregation in Germline Stem Cells. *Cell Stem Cell* **25**, 666-681 e665 (2019).
10. J. Cheng *et al.*, Centrosome misorientation reduces stem cell division during ageing. *Nature* **456**, 599-604 (2008).
11. Y. M. Yamashita, D. L. Jones, M. T. Fuller, Orientation of asymmetric stem cell division by the APC tumor suppressor and centrosome. *Science* **301**, 1547-1550 (2003).
12. Y. M. Yamashita, A. P. Mahowald, J. R. Perlin, M. T. Fuller, Asymmetric inheritance of mother versus daughter centrosome in stem cell division. *Science* **315**, 518-521 (2007).
13. S. Yadlapalli, J. Cheng, Y. M. Yamashita, Drosophila male germline stem cells do not asymmetrically segregate chromosome strands. *J Cell Sci* **124**, 933-939 (2011).
14. X. R. Sheng, E. Matunis, Live imaging of the Drosophila spermatogonial stem cell niche reveals novel mechanisms regulating germline stem cell output. *Development* **138**, 3367-3376 (2011).
15. M. Wooten *et al.*, Superresolution imaging of chromatin fibers to visualize epigenetic information on replicative DNA. *Nat Protoc* **15**, 1188-1208 (2020).
16. R. Cincinelli *et al.*, Novel adamantyl retinoid-related molecules with POLA1 inhibitory activity. *Bioorg Chem* **104**, 104253 (2020).
